# Regulation of SREBPs by Sphingomyelin in Adipocytes via a Caveolin and Ras-ERK-MAPK-CREB Signaling Pathway

**DOI:** 10.1371/journal.pone.0133181

**Published:** 2015-07-31

**Authors:** Nehman Makdissy, Katia Haddad, Charbel Mouawad, Iuliana Popa, Mohamed Younsi, Philippe Valet, Laurent Brunaud, Olivier Ziegler, Didier Quilliot

**Affiliations:** 1 Reviva Regenerative Medicine Center, Lebanese University—Faculty of sciences III, Middle East Institute of Health, Bsalim, Lebanon; 2 Laboratory of Nutrition & Metabolic Diseases, University Henri Poincaré - Nancy 1, Vandoeuvre les Nancy, France; 3 Laboratory of Dermatological Research, EA 4169 University Lyon-1, Lyon, France; 4 Institut National de la Santé et de la Recherche Médicale, U858, Toulouse, France; 5 Service de Diabétologie-Nutrition, CHU de Nancy, France; 6 INSERM 954, Faculty of Medicine, University Henri Poincaré - Nancy 1, France; UMR INSERM U866, FRANCE

## Abstract

Sterol response element binding protein (SREBP) is a key transcription factor in insulin and glucose metabolism. We previously demonstrated that elevated levels of membrane sphingomyelin (SM) were related to peroxisome proliferator–activated receptor-γ (PPARγ), which is a known target gene of SREBP-1 in adipocytes. However, the role of SM in SREBP expression in adipocytes remains unknown. In human abdominal adipose tissue from obese women with various concentrations of fasting plasma insulin, SREBP-1 proteins decreased in parallel with increases in membrane SM levels. An inverse correlation was found between the membrane SM content and the levels of SREBP-1c/ERK/Ras/PPARγ/CREB proteins. For the first time, we demonstrate the effects of SM and its signaling pathway in 3T3-F442A adipocytes. These cells were enriched or unenriched with SM in a range of concentrations similar to those observed in obese subjects by adding exogenous natural SMs (having different acyl chain lengths) or by inhibiting neutral sphingomyelinase. SM accumulated in caveolae of the plasma membrane within 24 h and then in the intracellular space. SM enrichment decreased SREBP-1 through the inhibition of extracellular signal-regulated protein kinase (ERK) but not JNK or p38 mitogen-activated protein kinase (MAPK). Ras/Raf-1/MEK1/2 and KSR proteins, which are upstream mediators of ERK, were down-regulated, whereas SREBP-2/caveolin and cholesterol were up-regulated. In SM-unmodulated adipocytes treated with DL-1-Phenyl-2-Palmitoylamino-3-morpholino-1-propanol (PPMP), where the ceramide level increased, the expression levels of SREBPs and ERK were modulated in an opposite direction relative to the SM-enriched cells. SM inhibited the insulin-induced expression of SREBP-1. Rosiglitazone, which is an anti-diabetic agent and potent activator of PPARγ, reversed the effects of SM on SREBP-1, PPARγ and CREB. Taken together, these findings provide novel insights indicating that excess membrane SM might be critical for regulating SREBPs in adipocytes *via* a MAPK-dependent pathway.

## Introduction

Numerous studies have revealed that sphingolipids are implicated in many diseases (inflammation, tumors, viral infections, and neurodegenerative diseases) and currently, sphingomyelin (SM) is considered an important sphingolipid and a major risk factor in the pathophysiology of atherosclerosis. Interestingly, SM may also play a role in metabolic syndrome and type 2 diabetes [1–4].

SM functions as a structural component of biological membranes, together with other phospholipids, glycolipids and cholesterol (CHOL). In addition to its structural role, increasing evidence suggests that SM affects major aspects of cellular functions, modulates the behavior of cellular proteins and receptors, and participates in signal transduction. Of the total cholesterol and sphingomyelin contents in the adipocyte plasma membrane, approximately 60% is found in the caveolae [5]. These domains are specialized transmembrane exchange zones implicated in cell signaling. SM is generated by SM synthase [6], which is thought to be the only enzyme that synthesizes SM in mammalian cells, and hydrolyzed by sphingomyelinase (SMase), generating ceramide [7–9]. SM metabolites are important cellular effectors and implicate SM in various cellular functions, such as development, differentiation, senescence and apoptosis [10–12]. Furthermore, SM may affect cellular signaling. Membrane SM was negatively related to the transcription factor peroxisome proliferator–activated receptor-γ (PPARγ) mRNA levels in subcutaneous adipocytes of obese insulin-resistant women [13] and in SM-enriched 3T3-F442A adipocytes [14]. Recently, adipose PPARγ has been identified as an essential mediator of lipid and glucose homeostasis and of whole body insulin sensitivity [8,15]. Several lines of evidence support roles for other transcription factors, in addition to PPARγ, in controlling adipogenesis and mediating insulin sensitivity; these transcription factors include the sterol response element binding proteins (SREBPs). In addition to their roles in insulin sensitivity, lipogenesis and lipid homeostasis, recent studies have revealed expanding roles for SREBPs in type II diabetes, cancer, immunity, neuroprotection, and autophagy [16].

SREBPs are a family of membrane-bound transcription factors that are composed of three subtypes, SREBP-1a, SREBP-1c and SREBP-2, which regulate the expression of multiple genes that play fundamental roles in both cholesterol and fatty acid metabolism and that are relevant to human diseases [16–18]. In addition to their regulation by metabolites and nutrients, these transcription factors are also targets of hormones (such as insulin and leptin), growth factors, inflammatory signals, and drugs. Major signaling pathways that couple transcription factors to extracellular stimuli include the mitogen-activated protein (MAP) kinase cascades through extracellular signal-regulated kinase (ERK1/2). In addition, SREBP-1 has been previously identified as a transcriptional regulator of caveolin expression in response to free CHOL. Caveolin, which is the major protein component of caveolae, is considered a caveolae marker; this protein is highly expressed in differentiated adipocytes and is central to the organization of proteins and lipids in caveolae and to the transport of CHOL to and from caveolae [19]. Caveolin functions as a negative regulator of ERK [20], and the intracellular activation of ERK has a direct stimulatory effect on the transcriptional activity of SREBP-1 [21].

The present study had two major objectives: 1) to determine the effect of excess membrane SM on SREBPs and 2) to identify whether the MAPK pathway mediates this relation. The consequences of this effect on CHOL and caveolin, as well as the role of ceramide, have also been assessed. Our findings indicate that elevated membrane SM negatively regulates SREBP-1 expression; the mechanism of action for SM seems to be dependent on the MAPK pathway *via* the down-regulation of Ras/KSR, Raf, MEK and ERK. Caveolin is regulated by elevated levels of SM, leading to changes in CHOL synthesis and in the regulation of the other isoforms of SREBP, including the positive regulation of CHOL-dependent SREBP-2. The present work also describes the SM-dependent down-regulation of SREBP-1/Ras/ERK/PPARγ/CREB expression in human adipose tissue.

## Materials and Methods

### Reagents and antibodies

DMEM, glutathione, and a bicinchoninic acid kit for protein determination were purchased from Sigma-Aldrich (Saint-Quentin Fallavier, France). 1,6-Diphenyl-1,3,5-hexatriene (DPH) was also obtained from Sigma-Aldrich (Saint-Quentin Fallavier, France). Sphingomyelins were obtained from Sigma-Aldrich or Matreya (PA, USA) or Avanti Polar Lipids (Alabama, Canada). GW4869 was obtained from Calbiochem VWR (Fontenay sous Bois, France). Fetal bovine serum was obtained from Eurobio (Les Ullis, France), and donor bovine serum was obtained from Institut Jacques Boy (Reims, France). The PCR primers were obtained from Proligo (Paris, France). The affinity-purified rabbit polyclonal SREBP-1 (H-160), SREBP-2 (N-19), ERK (K-23), p-ERK (E-4), p38 (H147), p-p38 (sc-17852), JNK (sc-571), p-JNK (sc-12882), MEK-1/2 (sc-436), p-MEK1/2 (sc-7995), Raf-1 (sc-133), p-Raf-1 (sc-16806), pan Ras (sc-14022), Ksr-1 (sc-25416), CREB-1 (sc-58), p-CREB-1 (sc-7978), PPARγ (H-100), caveolin-1 (N-20), nucleoporin p62 (H-122), and TGN38 (C-15) antibodies were obtained from Santa Cruz Biotechnology, Inc. (Santa Cruz, CA, USA). The caveolin-2 antibody (610684) was obtained from BD Transduction Laboratories (Lexington, KY, USA). The phospho-KSR-1 antibody (4951) was from Cell Signaling technology (Yvelines, france). Rosiglitazone was a gift from Dr. Laurent Brunaud and Prof. Oliver Ziegler. The SmartLaddermolecular weight marker for mRNA standard band size was obtained from Eurogentec (Angers, France). Protein mobility was compared with the following molecular weight markers: 21400–113000 (161–0305, Bio-Rad, Marnes La Coquette, France) and 2500–45000 (RPN755, Amersham Biosciences, Orsay, France). Polyvinylidene difluoride (PVDF) membranes for western blotting were purchased from Bio-Rad.

### Human adipose tissue collection

All clinical procedures were conducted according to the principles expressed in the Declaration of Helsinki, as revised in 2008 (http://www.wma.net/e/policy/b3.htm). All patients provided written informed consent, and samples were procured from Nancy University Hospital with Review Board approval from the Research Ethics Board (EC-CHU-Nancy) at the Centre Hospitalier Universitaire of Nancy (CHU-Nancy). Human subcutaneous adipose tissue samples were obtained from 23 morbidly (grade III) obese women [40.0±9.3 yrs old, body mass index: 47.3±6.8 kg/m^2^] before bariatric surgery. The tissue samples were immediately processed for RNA extraction or lipid extraction, frozen in liquid nitrogen, or stored at –80°C. None of the subjects had been involved in a weight reduction program over the previous period of 12 months. The glucose tolerance of the patients, based on the oral glucose tolerance test, was normal according to World Health Organization criteria. None of the subjects was treated with any drug that could influence plasma lipid concentrations or glucose tolerance. The subjects were: Normotensive [with diastolic and systolic blood pressure inferior to 90 and 140 mmHg, respectively]; Normocholesterolemic [total serum cholesterol (< 250 mg/dL), LDL cholesterol (< 150 mg/dL), HDL cholesterol (> 40 mg/dL)]; Normotriglyceridemic (< 200 mg/dL); with fasting glucose (< 120 mg/dL).

### Cell culture and differentiation

3T3-F442A fibroblasts were cultured in DMEM containing 25 mM glucose and 0.1 g/L gentamycin, supplemented with 10% (v/v) donor bovine serum at 37°C in 7% CO_2_. Two days after the cells reached confluence, differentiation was induced using DMEM containing 50 nM insulin and supplemented with 10% fetal bovine serum. The medium was changed every 2 days. Insulin was removed 2 days before the experiments, and the culture medium was replaced with DMEM containing 5 mM glucose for 24 h before the experiments. The cells were used 10 days after the induction of differentiation when more than 85–90% of the cells had an adipocyte-like phenotype.

### Preparation of total and crude plasma membranes

The medium for the 3T3-F442A adipocytes in 100-mm dishes was removed and replaced with serum-free DMEM for 2 h. After 2 washings with ice-cold PBS, the cells were incubated with 3 ml of homogenization buffer (1 mM EDTA, 10 mM Tris-HCl, and 1 mM phenylmethylsulfonyl fluoride, pH 7.4) for 5 min and then pipetted off the dishes. The cell suspension was homogenized by 10 passages through a 26-gauge needle and resuspended in 18 ml of ice-cold homogenization buffer. Cell disruption was monitored under a microscope. The homogenate was centrifuged at 250000×*g* (Kontron TFT65.38 rotor) for 90 min at 4°C to pellet down the total membranes [22]. To obtain the crude plasma membranes, the homogenate was centrifuged at 8500×*g* (Beckman JA17 rotor) for 10 min at 4°C, and the supernatant was centrifuged twice at 40000×*g* (Kontron TFT65.38 rotor) for 30 min at 4°C [23]. Supernatants were removed and the pellets (membrane fraction) containing total or crude plasma membranes were resuspended in 1 ml of buffer (1 mM EDTA, 1 mM Tris-HCl, and 10 mM NaCl, pH 7.4). A 50 μl aliquot was used for the determination of protein content, and the membrane samples were kept at –80°C until used.

### Preparation of caveolae*/*lipid rafts

Caveolae/lipid rafts were prepared according to the method of Smart *et al*. [24]. Briefly, differenciated adipocytes were pelleted and resuspended in L-buffer (0.25 M sucrose, 20 mM tricine, and 1 mM EDTA, pH 7.8) containing protease inhibitors and Dounce-homogenized (25 strokes). Cell debris and nuclei were removed by centrifugation at 1000×*g* for 12 min. After resuspension of the resulting pellet in L-buffer and centrifugation, the supernatants were combined (called the post-nuclear supernatant (PNS)) and loaded onto 25 ml of 30% (w/v) Percoll in L-buffer before centrifugation at 26000 rpm (Beckman SW28 rotor) for 45 min. The plasma membrane (PM) fraction (a visible band 5–5.6 cm from the bottom of the centrifuge tube) was collected and sonicated (3×20 s). The resulting sonicate was mixed with 50% (w/v) Opti-Prep in P-buffer (0.25 M sucrose, 120 mM tricine, and 6 mM EDTA, pH 7.8) containing protease inhibitors to yield 4 ml of 23% Opti-Prep and loaded at the bottom of a centrifuge tube. Then, 6 ml of a 10–20% (w/v) gradient was layered on top and the sample was centrifuged at 22000 rpm—for 90 min. The resulting non-floating fraction (called the non-caveolae membrane/non-lipid raft (NCM) fraction) was collected from the bottom ~4 ml of the tube). The floating fraction (top ~5 ml of the tube) was collected, mixed with 4 ml of 50% (w/v) Opti-Prep, and loaded at the bottom of another tube. Then, 2 ml of 5% (w/v) Opti-Prep was layered on top of this, and the sample was centrifuged at 22000 rpm-for 90 min. The light-scattering band (called the standard caveolae membrane/lipid raft (CM) fraction) was collected directly above the interface of these two gradients. The recovery of caveolin in the caveolae fraction was 29.6±3.2% (mean±SEM, *n* = 3 independent preparations), as determined by immunoblotting. The nuclear fraction was obtained by resuspending and centrifuging the 1000×*g* pellet through a cushion of 1.6 M sucrose at 100000×*g* for 35 min. The microsomal fraction was obtained by pelleting the supernatant (obtained from the 16000×*g (26000 rpm)* centrifugation) at 200000×*g* for 75 min.

To ensure the absence of nuclear and microsomal membrane contamination in the plasma membrane fraction, we analyzed this fraction: (1) for the presence of the nuclear protein nucleoporin p62 using the antibody H-122 (sc-25523, Santa Cruz Biotechnology, Inc.), which is a rabbit polyclonal antibody raised against amino acids 401–522 of human nucleoporin p62; and (2) for the presence of the largely Golgi-localized protein TGN38 using the antibody C-15 (sc-27680, Santa Cruz Biotechnology, Inc.), which is a goat polyclonal antibody raised against a peptide mapping within a cytoplasmic domain of human TGN38. The appearance of this protein indicated a low level of Golgi contamination of the plasma membrane fraction because this *trans*-Golgi network protein cycles between the Golgi and the plasma membrane.

### Isolation of triglyceride droplets (TGD)

Differentiated adipocytes were washed twice with ice-cold PBS and gently homogenized in hypotonic PBS containing protease inhibitors. TGD were isolated by ultracentrifugation (Beckman SW-41 rotor) at 100,000×*g* in T-buffer (0.25 M sucrose, 50 mM Tris-HCl, pH 7.6, 5 mM MgCl2, and 25 mM KCl) for 60 minutes at 4°C. After collection of the supernatant fraction (containing TGD), the pellet fraction was washed twice with T-buffer (Beckman TLA-120.2).

### Addition of exogenous sphingomyelins (SMs), sphingomyelinase inhibitors (glutathione and GW4869) or DL-threo-1-phenyl-2-palmitoylamino-3-morpholino-1-propanol (PPMP)

The adipocytes were treated with SMs, GSH, GW4869 and PPMP for 24 h before cell harvesting, and control cells received equal amounts of vehicle. PPMP, which was dissolved in ethanol at 37°C (3 mM), was added to DMEM (20 μM). Glutathione (GSH), which was dissolved in water (200 mM), was added to DMEM (10 mM). GW4869, which was dissolved in Me_2_SO/MSA-H_2_O (1.43 mM), was added to DMEM (10 μM) and routinely stored at -80°C as a 1.5 mM stock suspension in Me_2_SO. Immediately before use, this stock suspension was solubilized by the addition of 5% methane sulfonic acid (MSA) (2.5 μl of 5% MSA in sterile double-distilled H_2_O was added to 50 μl of the GW4869 stock suspension). Then, the suspension was mixed and warmed at 37°C until clear, and the concentration of the GW4869 stock solution at the time of the experiments was 1.43 mM. Four exogenous SMs were used: (i) natural: SM-PA (primarily with palmitic acid), SM-SNA (primarily with stearic and nervonic acids) and SM-LA (primarily with lignoceric acid) and (ii) synthetic: *syn*-SM (N-lignoceroyl-D-*erythro*-sphingosylphosphorylcholine). SMs were dissolved in (1) water, (2) ethanol or (3) ethanol/dodecan (98/2, v/v) at room temperature or at 37°C. After dissolution, the cells were treated with SMs (15 μM, 24 h). Exogenous SMs were insoluble in water and aggregated; however, the viability of the cells was not affected, and no changes in the levels of membrane SM occurred. Ethanol partially dissolved the SMs at room temperature; however, better efficacy was observed when the ethanol was pre-warmed to 37°C. The viability of the cells was not affected; nevertheless, significant changes occurred at the level of membrane SM. Finally, although ethanol/dodecan was used at room temperature or warmed at 37°C, the SMs greatly accumulated in the plasma membrane (reaching a 2.1-fold increase). However, significant decreases in the viability of the cells were detected [viability ranged from 48–59% (solvent at room temperature) or from 8–21% (solvent at 37°C)]. In the case of control cells (vehicle-treated), notably, ethanol/dodecan was also toxic under all tested conditions, at room temperature or at 37°C, with viability values of 62% and 46%, respectively. Considering these results, SMs were systematically dissolved in absolute ethanol at 37°C, added to DMEM [ethanol to medium ratio: 1/200 (v/v)] and kept at 37°C for 10 min for all experiments described in this work. Then, standard media were removed from the culture flasks and replaced by media containing SM.

### Membrane phospholipids and cholesterol determination

Lipids were extracted from cell total membranes or from plasma membranes using methanol and chloroform (11:7, v/v) according to the method of Rose and Oklander [25]. The amount of membrane proteins used for optimal extraction was 500 μg. The organic phase was evaporated to dryness under a stream of nitrogen at room temperature. The lipid residue was dissolved in chloroform, and its components were separated by HPLC on a Gold liquid chromatographic system equipped with a Model 126 pump and a Model 406 interface (Beckman, Palo Alto, CA, USA), which was monitored using an IBM microcomputer (IBM, Courbevoie, France). Twenty micrograms of lysophosphatidylcholine per 100 μg proteins was added as an internal standard before the extraction. The major classes of phospholipids (phosphatidylcholine (PC), phosphatidylethanolamine (PE), phosphatidylserine (PS), phosphatidylinositol (PI), and sphingomyelin (SM)) were detected [13] using an evaporative light scattering detector (Sedere, Orleans, France). Quantification was based on the comparison of the integrated peak area using curves prepared from standard phospholipid solutions. Total SM was used; the type of fatty acid coupled had no influence on SM identification and quantification. In fact, PL separation occurs in two phases: the first phase is mobile (methanol and chloroform), and the second phase is separated on a column; however, the elution is performed only based on the polarity and not based on the length or type of the fatty acid constituting the SM. CHOL was quantified by the method of Zlatkis and Zak [26].

### Purification of sphingolipids

Sphingolipids (ceramides and glycolipids) were isolated on LC-NH2 cartridges (Supelco) according to Popa *et al*. [27] using a modification of the method of Bodennec *et al*. [28]. The total lipids were taken up with 1 ml of diethylether and applied on a 3 ml LC-NH2 cartridge. Neutral lipids were eluted with 4 ml of diethylether, and then ceramides were eluted with 4 ml of chloroform-methanol 23:1 (v/v). After the elution of free fatty acids with 3 ml of di-isopropylether-acetic acid 98:5 (v/v), neutral glycolipids were eluted with 4 ml of acetone-methanol 9/1.4 (v/v). The ceramide and neutral glycolipid fractions were evaporated under nitrogen and taken up with chloroform-methanol 2:1 (v/v). An aliquot of each fraction was analyzed by thin-layer chromatography on silica gel plates (HPTLC plates, Merck); ceramides migrated in chloroform-methanol 97:3 (v/v), and neutral glycolipids migrated in chloroform-methanol-water 65:25:4 (by volume). After drying, the plates were visualized by spraying 3% copper acetate in 8% phosphoric acid and heating at 150°C for 5 min. The sphingolipid (ceramides and glucosylceramides) assay was performed by fluorescence with fluorescamine (Sigma-Aldrich) after the acid hydrolysis of sphingolipids according to Naoi *et al*. [29], using sphingosine (Sigma) as a standard.

The possible presence of galactosylceramide was investigated by thin-layer chromatography on borate-impregnated silica gel plates according to Kean [30]. A thin-layer silica gel plate was sprayed with 1% sodium borate in methanol, and then the plate was dried at 120°C. After spotting a sample of the neutral glycolipid fraction along with standard galactosylceramide from bovine brain (Sigma-Aldrich), the plate was developed in chloroform-methanol-water-28% ammonia 40:10:0.9:0.1 (by volume). After drying, the plate was visualized with an orcinol-sulfuric acid spray reagent at 120°C. Notably, galactosylceramide was not detected in the extracts.

### Filipin staining of free cholesterol and fluorescence microscopy

Differentiated cells on polylysine-coated slides were treated with SMs or vehicle for 24 h. Filipin staining was performed essentially as previously described [31]. The adipocytes were viewed with a fluorescence microscope (Axioskop, Zeiss) using a UV filter set (365-nm excitation, 395-nm beam splitter and 420-nm long pass filter). The microscope was coupled with a camera (Hamamatsu Photonics C5310, Japan) and with SigmaScan Pro software (SPSS, Erkrath, Germany) to capture images.

### Membrane fluidity

The membrane fluidity was assessed by steady-state fluorescence polarization using the lipophilic fluorescent probe 1,6-diphenyl-1,3,5-hexatriene (DPH). The fluorescence anisotropy of this probe, which is inversely related to fluidity, was determined as previously described [32].

### Determination of gene expression by RT-PCR

The media were removed 24 h after treatments, and the cells were washed with cold PBS. Total cellular RNA was prepared using an RNeasy Mini Kit (Qiagen, Courtabeuf, France) according to the manufacturer’s instructions. DNase digestion was performed in parallel to ensure the absence of genomic DNA contamination using a RNase-Free DNase Set from Qiagen. The yield and purity of total RNA were measured spectrophotometrically. The A260/280 ratio ranged from 1.85–2 for all preparations. RNA integrity was verified by agarose gel electrophoresis. First-strand cDNA was synthesized (Bio-Rad iCycler thermal cycler) with the Super Script RNase H Reverse Transcriptase (Invitrogen, Cergy Pontoise, France) in the presence of random hexamer primers using 2 μg of total RNA in a 20 μl final volume. β-Actin was used as the housekeeping gene. The primers and PCR conditions are shown in Table 1. For semi-quantitative PCR, duplex PCR reactions were performed in 50 μl final volumes, separated on a 2% agarose gel containing 0.5 μg/ml ethidium bromide and detected by transillumination using UV light (Gel Doc 2000 imaging system, Bio-Rad). The MW-1700-02 SmartLadder molecular weight marker (Eurogentec) was used as a standard for band size. PCR analyses were performed using a Bio-Rad iQ Cycler. For quantitative real-time PCR, PCR reactions using cDNA equivalent to 100 ng of total RNA for SREBP-1 and 10 ng for the other genes were performed in 15 μl final volumes using the iQ SYBR Green Supermix (Bio-Rad). The relative quantification of gene expression was determined using the comparative Ct method (R = 2^-ΔΔCt^). A fold change ≥2 indicated the up-regulation of a gene. No variation was detectable when 0.5≤R≤2. A fold change ≤0.5 indicated the down-regulation of a gene and was expressed as a negative number (i.e., a fold change of 0.5 was expressed as -2.00).

**Table 1:** Primers and polymerase chain reaction (PCR) conditions. *(A) Non-quantitative PCR*: the first step of the PCR reaction was at 94°C for 5 min to activate the polymerase, followed by the indicated number of cycles of denaturation at 94°C for 45 s, annealing for 60 s at the indicated temperature for each gene, extension at 72°C for 90 s and an additional extension at 72°C for 5 min after the last cycle. *(B) Real-time PCR*: the first step was 95°C for 3 min, followed by 45 cycles of denaturation (95°C for 10 s), annealing (10 s at the indicated temperature for each gene) and extension (72°C for 10 s).

Gene name	Primer	Sequence (5′→ 3′)	Product size (bp)	PCR conditions	
				Annealing temperature in°C	Cycle number
β-actin*	sense/ antisense	GGTCGTACCACAGGCATTGTGATG/ GGAGAGCATAGCCCTCGTAGATGG	78	Identical to the studied gene	Identical to the studied gene
***A) Non-quantitative PCR***					
SREBP-1	sense/ antisense	ACCATCGGCACCCGCTGCTTTAAAGAT/ TGAATGGTGGCTGCTGAGTGTTTCCTG	253	63	35
SREBP-2	sense/ antisense	CACAATATCATTGAAAAGCGCTACCGGTCC/ TTTTTCTGATTGGCCAGCTTCAGCACCATG	200	63	35
Caveolin-1	sense/ antisense	AGCATGTCTGGGGGCAAATAC/ CTTGACCACGTCGTCGTTGAG	198	62	35
Caveolin-2	sense/ antisense	TGAGAAGTATGTTGACTCGAGTC/ GCAAACAGGATACCCGCAATGAA	224	66	35
***B) Real-time PCR***					
SREBP-1	sense/ antisense	CTGGCTTGGTGATGCTATGTTGAG/ AGGGCTGGAAGGCAAAGGAAC	105	58	45
SREBP-2	sense/ antisense	TGGGCATGGTGGACCGCTCTC/ TGGGTGCTGGTCATGGTTGTGGG	119	60	45
Caveolin-1	sense/ antisense	AGCATGTCTGGGGGCAAATAC/ CTTGACCACGTCGTCGTTGAG	198	60	45
Caveolin-2	sense/ antisense	CGATGACGCCTACAGCCACCACA/ TGGTGAGGATCCCGGTCGTGACT	87	60	45

* Primers for β-actin were used for both non-quantitative and real-time PCR.

### Western blot analysis

Cells were solubilized in 0.5 ml of cold lysis buffer [10 mM Tris-HCl, 150 mM NaCl, and 5 mM EDTA (pH 7.4) containing 1 mM phenylmethylsulfonyl fluoride, 1%(w/v) Triton X-100, 60 mM octyl β-D-glucopyranoside and cocktails of phosphatase and protease inhibitors] and centrifuged at 15000×*g* for 20 min at 4°C. After fat elimination, the supernatants were used for protein determination and western blot analysis. Total and crude plasma membrane fractions were prepared as described above. Nuclear and cytoplasmic protein extracts were prepared using a CelLytic NuCLEAR Extraction Kit from Sigma-Aldrich (N-XTRACT) according to the manufacturer’s instructions. One volume of cellular/nuclear lysate or total or crude plasma membrane fractions was mixed with one volume of sample buffer (125 mM Tris-HCl (pH 6.8), 4% SDS, 20% saccharose, 4% mercaptoethanol, and 0.0005% bromophenol blue) and frozen at –80°C for later immunoblotting. Before SDS-PAGE analysis, dithiothreitol was added (final concentration: 150 mM), and the samples were heated at 95°C for 10 min. SDS-PAGE analysis was performed on a resolving gel (7.5% for SREBPs, Raf/p-Raf and KSR/p-KSR; 10% for ERKs/p-ERKs, MEK/p-MEK, p38/p-p38, JNK/p-JNK, CREB/p-CREB and PPARγ; and 12% for Ras and caveolins) and on a 4% stacking gel. Then, the proteins were transferred onto a PVDF membrane using a Bio-Rad Mini Trans-Blot apparatus and detected using a Protein Detector LumiGLO Western Blot Kit (54-12-50, KPL Laboratories, Gaithersburg, MD, USA) according to the manufacturer’s instructions. The membranes were probed with antibodies directed against SREBP-1 and SREBP-2 (1/200 and 1/400, respectively, 1 h incubation), ERK/p—ERK (1/1000 and 1/300, respectively, overnight incubation), Ras (1/1000, 1 h incubation), Raf/p-Raf and Mek1/2-p-MEK1/2 (1/500 each, 1 h incubation), KSR/p-KSR (1/400, 1 h incubation), caveolin-1/2, nucleoporin p62 and TGN38 (1/250, 1 h incubation). Bound antibodies were visualized by incubation with a peroxidase-conjugated anti-immunoglobulin G polyclonal antibody. Equal protein loading in all experiments was confirmed by Coomassie blue staining of the blots. Protein mobility was compared using molecular weight markers. BSA (5 μg) was used as an internal standard. Western blotting luminol reagent (Santa Cruz Biotechnology, Inc.) was used to visualize the antigen-antibody complex. The intensities of the bands were quantified using a Gel Doc 2000 imaging system and Quantity One software (Bio-Rad).

### Ras Activation ELISA Assay

The Ras activity was assessed by using the Ras Activation ELISA Assay Kit (Millipore, Fontenay-sous-Bois, France) following the instructions of the manufacturer. Briefly, the assay works on the principle that Ras only binds to its downstream kinase, Raf-1 (MAP Kinase Kinase Kinase), when in its active-GTP bound state. In this state, Ras binds to a domain of Raf-1 kinase referred to as the Ras Binding Domain (RBD). Via a GST/Glutathione interaction with a 96-well gluthatione-coated ELISA plate, a recombinant Raf-1-RBD captures the activated/GTP-bound Ras and allows the inactive/GDP-bound Ras to be washed away. The captured active Ras is detected and measured quantitatively through the addition of a monoclonal anti-Ras antibody that detects K-, H-, N- Ras isoforms. An HRP conjugated secondary antibody was then added for the detection after the addition of the chemiluminescent substrate. Signals were measured using a CCD camera (Bio-Rad).

### Statistical analysis

The effects of different SMs, GSH or PPMP were expressed relative to the controls, which were assigned an arbitrary value of 100 or 1. The results are presented as the means±SEM and have been analyzed for statistical significance using ANOVAS tests, followed by Student-Newman-Keuls tests (StatView software, SAS Institute, Cary, NC, USA). Univariate statistical analysis was performed by linear regression analysis to indicate correlations between the variables. The level of significance was set at *P*<0.05.

## Results

### SREBPs and sphingomyelin in human adipose tissue

**Fig 1:**
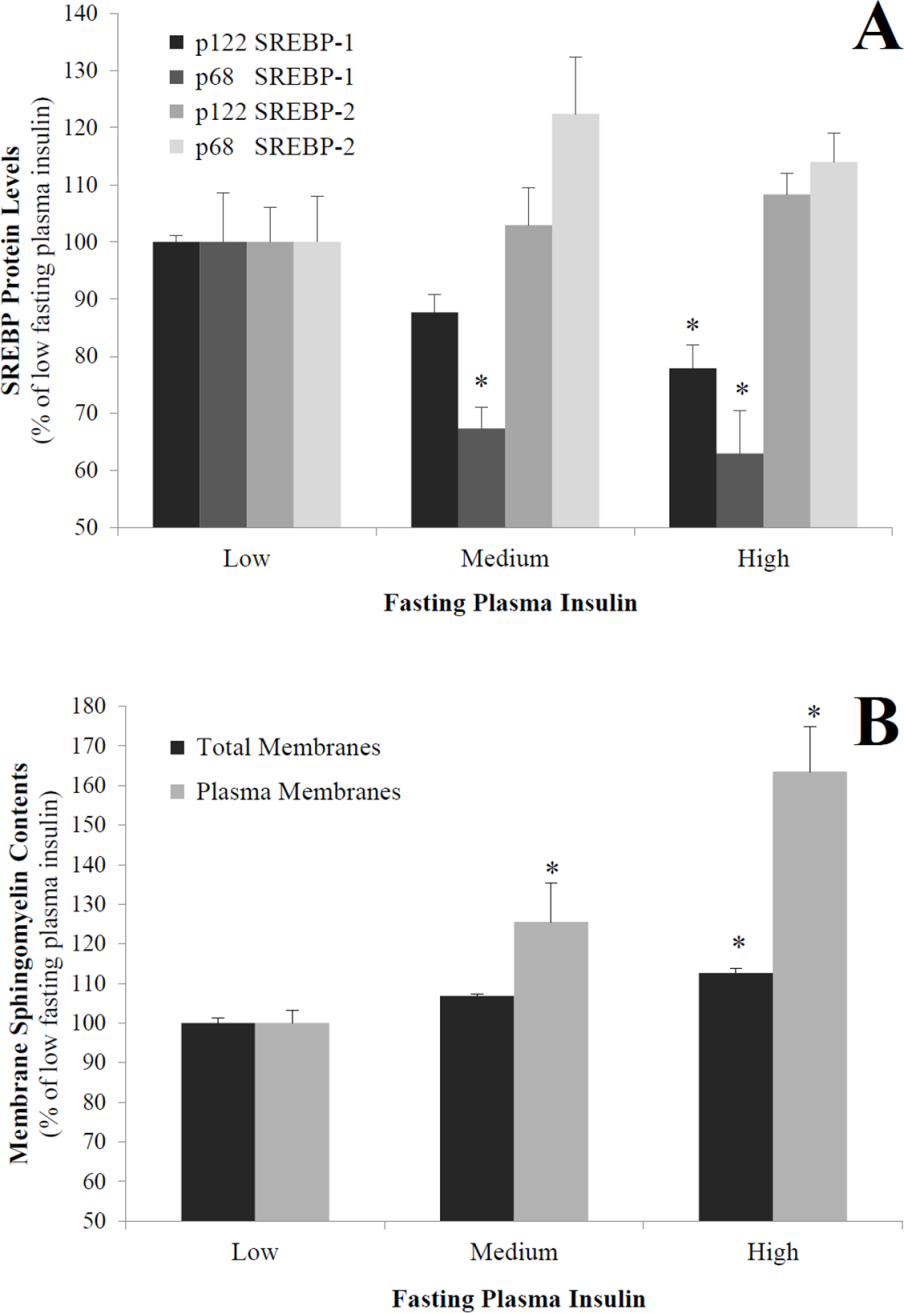
Levels of SREBP proteins (A) and sphingomyelin in total and plasma membranes (B) in human adipose tissue. In total, 23 obese women were grouped into tertiles according to their fasting plasma insulin (FPI) concentrations. Mean FPI±SEM (mU/L): low, 10.5±2.1; medium, 17.6±1.3; and high, 36.2±4.1. The results are expressed as percentages of low FPI cells. Cell lysates were separated by SDS-PAGE and immunoblotted with antibodies reacting with both precursor (p122) and cleaved active (p68) proteins of SREBP-1 or SREBP-2 (100 μg of protein). Total and plasma membranes were prepared, and sphingomyelin concentrations were determined as described in the Materials and methods. ^*^ *P*<0.05; high or medium FPI compared with low FPI.

First, the fasting plasma insulin (FPI) was evaluated in the group of 23 obese women and displayed significant variations (8.4–40.3 mU/L). Then, these subjects were grouped into tertiles according to FPI: low, 10.5±2.1 mU/L (n = 8); medium, 17.6±1.3 mU/L (n = 7); and high, 36.2±4.1 mU/L (n = 8). Subjects in the highest FPI tertile had significantly lower levels of SREBP-1 proteins [(p122: -22.1%; *P*<0.05), (p68: -37.0%; *P*<0.05)] in contrast to SREBP-2, where the differences did not reach significance (Fig 1A). In addition, subjects with the highest FPI had increased levels of sphingomyelin in their total (12.6%, *P*<0.05) and plasma (63.5%, *P*<0.05) membrane samples (Fig 1B).

### Profile of expression of SREBP-1c, RAS, ERK, CREB and PPARγ and membrane SM in human adipose tissue

**Fig 2:**
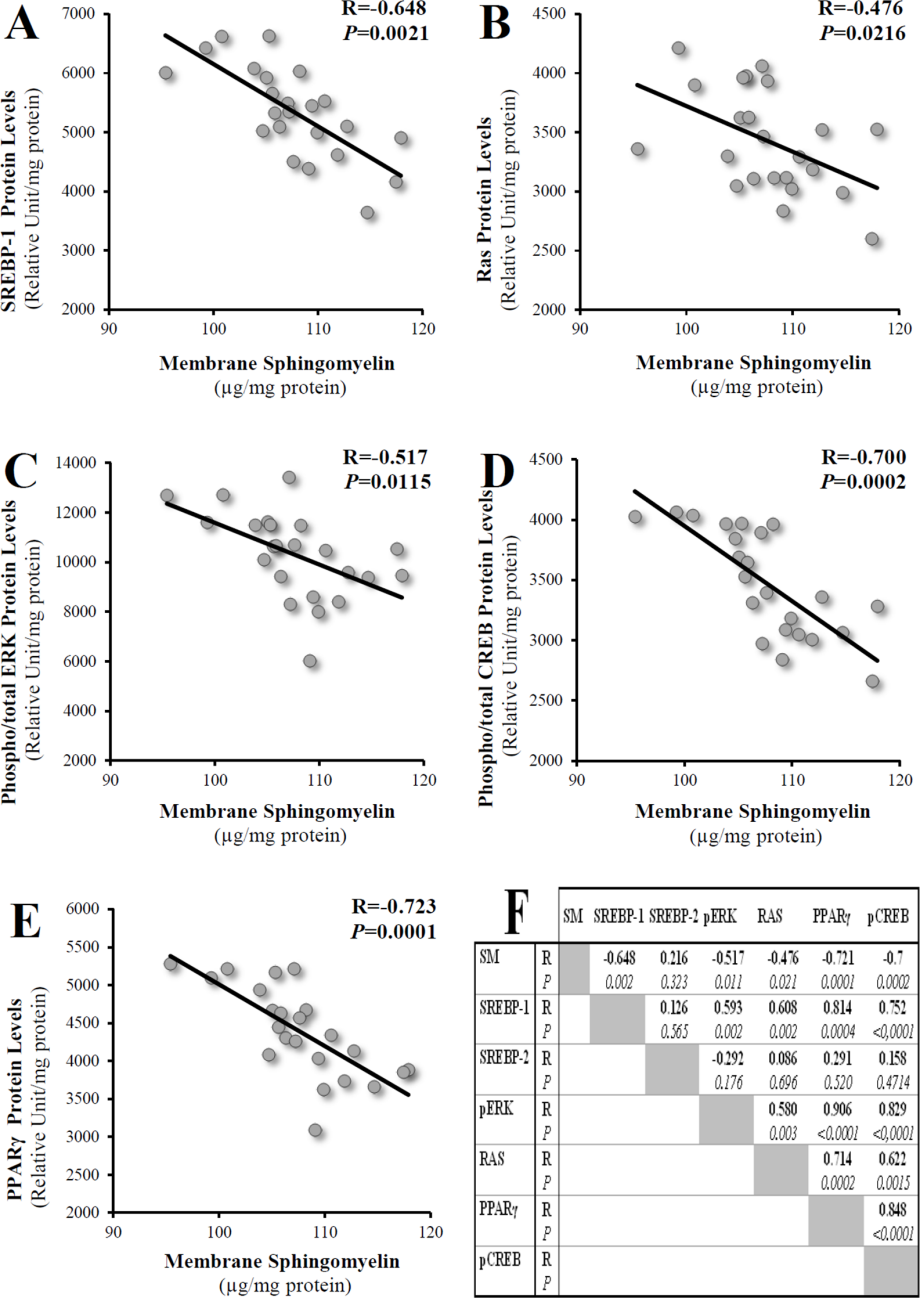
Relations between SM accumulation and SREBP-1, Ras, ERK, CREB and PPARγ proteins in human subcutaneous adipose tissue. R, coefficient of correlation; statistical significance was set at *P*<0.05. Membrane SM levels refer to total membrane levels.

We examined whether any correlation existed between SM levels and the following other potentially involved genes and proteins: CREB, PPARγ and MAPK. Negative linear correlations were found between total membrane SM levels and SREBP-1c mRNA (R = -0.51, *P* = 0.036), p68 SREBP-1 protein (R = -0.648, *P* = 0.002), Ras protein (R = -0.476, *P* = 0.021), phospho/total ERK proteins (R = -0.517, *P* = 0.011), phospho/total CREB proteins (R = -0.7, *P* = 0.0002), and PPARγ proteins (R = -0.721, *P* = 0.0001) (Fig 2). No significant correlations were found with SREBP-2 proteins. Note that SREBP-1 positively correlated with Ras (R = 0.608, *P* = 0.002), p-ERK (R = 0.593, *P* = 0.002), PPARγ (R = 0.814, *P* = 0.0004) and p-CREB (R = 0.752, *P*<0.0001) proteins. Using this same group of human adipose tissue samples, significant correlations were previously reported for the LRP1 receptor and adipogenesis and for TNF-α and apelin expression [33,34].

### Sphingomyelin enrichment in 3T3-F442A adipocytes

To evaluate the effect of SM on the expression of SREBPs and to determine its mechanism of action, we addressed the second part of the study *in vitro* using 3T3-F422A adipocytes. Two cellular models of membrane enrichment with SM were investigated by treating mature adipocytes with different exogenous SMs [(i) first natural: SM-PA (primarily C16:0), SM-SNA (primarily C18:0 and C24:1), SM-LA (primarily C24:0) and (ii) synthetic: syn-SM (C24:0)] [35–38] and second by inhibiting neutral sphingomyelinase with glutathione GSH [39]. In parallel, cells enriched with ceramide, which is the major metabolite of SM, were also studied using treatment with PPMP, which is an inhibitor of glucosylceramide synthase [40], increasing intracellular ceramide levels.

The initial observations showed no loss of adhesion of 3T3-F442A adipocytes or cell viability, which was greater than 98% in the cases of exogenous SMs dissolved in prewarmed ethanol (as indicated in the Materials and methods), regardless of the time course of the cell treatment and the SM concentrations (up to 240 μM), as determined by trypan blue. However, the exposure of adipocytes to GSH (≥20 mM) or PPMP (≥50 μM) for 24 h induced significant cell death; therefore, all the experiments were conducted with 10 mM of GSH but with 20 μM of PPMP (cell viability: >98%).

**Fig 3:**
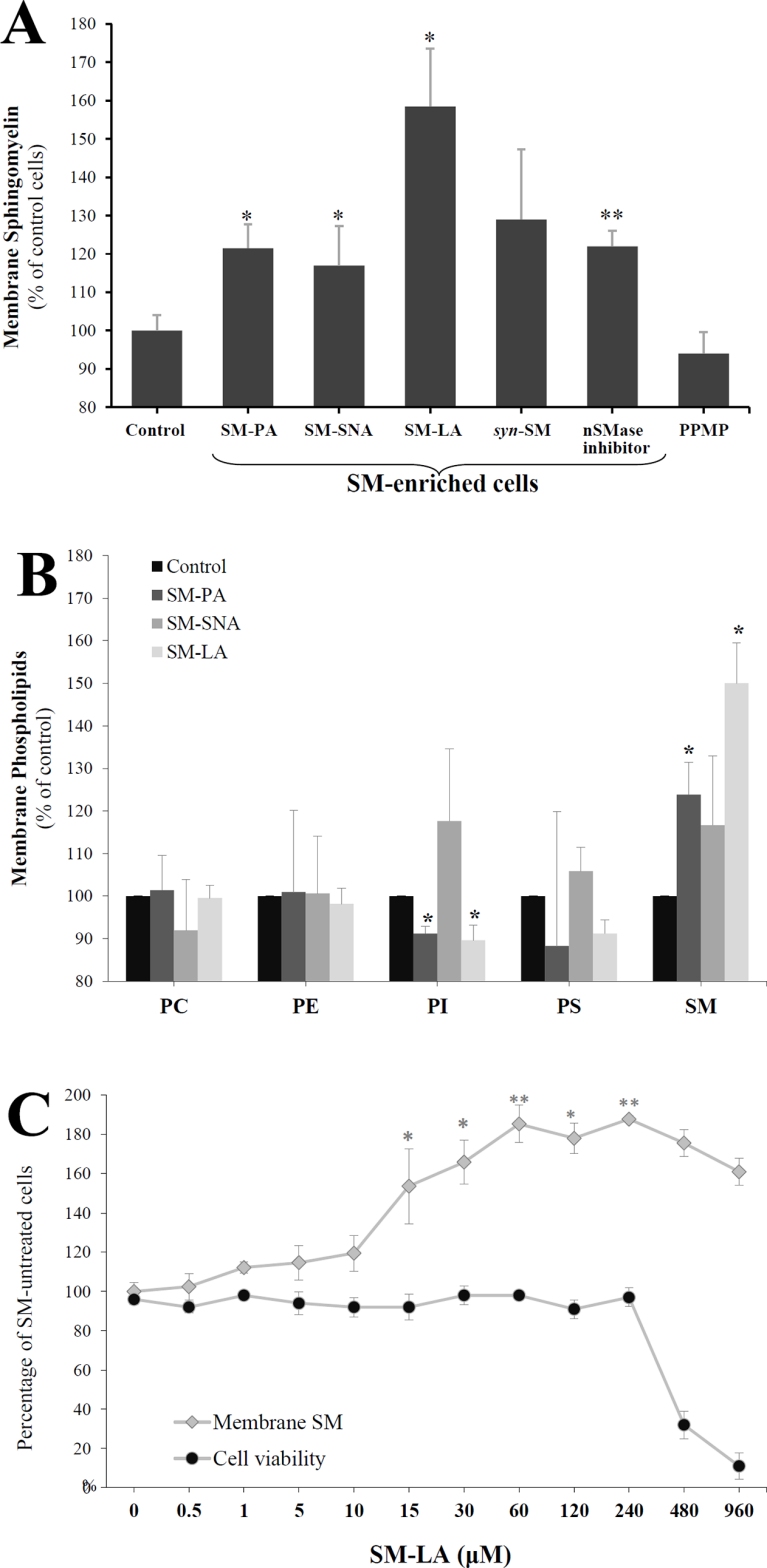
Changes in the membrane phospholipid contents of 3T3-F442A adipocytes. On day 9 of differentiation, the culture medium was supplemented with or without 15 μM exogenous SMs, 10 mM GSH (N-SMase inhibitor) or 20 μM PPMP for 24 h. Control cells were incubated with vehicle. The effects of four exogenous SMs were determined: (i) three natural with different acyl chains, primarily palmitic acid (SM-PA), stearic and nervonic acids (SM-SNA), or lignoceric acid (SM-LA), and (ii) one synthetic, namely, N-lignoceroyl-D-*erythro*-sphingosylphosphorylcholine (*syn*-SM). Total and plasma membranes were prepared, and phospholipid concentrations were determined as described in the materials and methods. (A) Levels of sphingomyelin in total membranes. (B) Levels of major phospholipids in plasma membranes following 24 h (15 μM) incubation with SM-PA, SM-SNA and SM-LA. (C) Dose-response relation. The cells were treated for 24 h with SM-LA (0–960 μM) and the levels of sphingomyelin in plasma membranes were quantified. The results are expressed as μg of the studied phospholipid per mg protein, presented as percentages of control cells, and are the mean±SEM of three independent experiments, which were each performed in triplicate (A) or duplicate (B and C). ^*^ *P*<0.05; SM-treated cells compared with control cells. ^**^ *P*<0.01; SM-, GSH-, and PPMP-treated cells compared with control cells. SM, sphingomyelin; PI, phosphatidylinositol; PS, phosphatidylserine; PE, phosphatidylethanolamine; PC, phosphatidylcholine.

First, the sphingomyelin levels in total membranes were determined (Fig 3A). The sphingomyelin level in control adipocytes was 29±2 μg/mg protein. PPMP-treated adipocytes displayed moderate decreases in the membrane sphingomyelin level that did not reach the level of significance (-6%, NS). However, the SM levels increased by 23% (*P*<0.01) in the total membrane fraction of adipocytes treated with the SMase inhibitor GSH. The treatment with exogenous SMs (15 μM, 24 h) induced the following significant accumulations of SM in total membranes: 35±2 (21%), 34±3 (17%) and 46±7 (59%) μg/mg protein for SM-PA, SM-SNA and SM-LA, respectively. Expressing the results in terms of the percentage of total phospholipids also indicated significantly increased levels of SM in membranes in the same order (SM-LA>SM-PA>SM-SNA: 40, 19 and 15%, respectively). Synthetic exogenous SM treatment (*syn*-SM) induced an increase that did not reach the level of significance (29%, NS).

Next, we evaluated whether the plasma membrane was affected by SM treatments. Then, the major phospholipid contents were determined in plasma membranes (Fig 3B). PC represented the major component (43.4%±3.1), followed by PE, PI, PS and SM (33.1±2.6, 12.5±1.4, 6.8±1.6 and 4.2±0.2, respectively) in control cells. The SM levels increased by 24% (*P*<0.05), 17% (NS) and 50% (*P*<0.05) after treatment with 15 μM (24 h) of SM-PA, SM-SNA and SM-LA, respectively, in contrast to the PI levels, which decreased significantly in the cases of SM-PA (-9%, *P*<0.05) and SM-LA (-10%, *P*<0.05) treatments. No significant change occurred in the other phospholipids classes. Interestingly, the range of membrane SM variations (from 17 to 59%) in SM-enriched cells was similar to that observed in adipocyte membranes of insulin-resistant obese patients compared with those of insulin sensitive patients, reproducing the situation found in human insulin resistance [41].

In the second set of experiments, the dose-response relation of the SM treatment was evaluated. Adipocytes were treated with SM-LA (0–960 μM, 24 h). The levels of sphingomyelin were determined in the plasma membrane, and cell viability was assessed. The results (Fig 3C) indicated an accumulation of sphingomyelin in the plasma membrane that appeared to be dose-dependent (range of significant increases: 54–88%); however, this treatment was toxic at 480 and 960 μM concentrations (the percentages of cell viability were 32 and 11%, respectively). The range of cell viability at concentrations less than or equal to 240 μM was 92–98%. Thus, the dose-response relation was limited to the SM-LA (24 h) treatment with concentrations less than or equal to 240 μM.

### Subcellular distribution of sphingomyelin

To determine the subcellular distribution of sphingomyelin after treating 3T3-F442A adipocytes with exogenous SM-LA (15 μM), the cells were treated for 2, 4, 8, 24, 32 and 48 h. The viability of the cells was not affected by this treatment (>96%). To ensure that the plasma membrane fraction was not contaminated with nuclear and microsomal membranes, we analyzed the samples for the presence of the nuclear protein Nucleoporin p62 and of the primarily Golgi-localized protein TGN38, respectively. The results (Table 2) indicated that the Nucleoprotein p62 and TGN38 proteins levels were 0.9% and 3.8% in plasma membrane samples relative to the nuclear and microsomal levels, respectively. The data (Table 3) demonstrated that SM accumulates in the PM within 24 h and then in the intracellular space. Interestingly, after 24 h of SM-LA incubation, although the NCM and CM were both derived from the PM, SM was greatly enriched in the CM (~6.5-fold) compared with the NCM, in which a slight increase was detected (~1.2-fold); however a significant increase was detected in the PNS (~1.7-fold). After 48 h of treatment, sphingomyelin significantly accumulated in the PNS (~7.3-fold), in contrast to the caveolae membranes, where SM levels declined (reaching ~1.3-fold).

**Table 2:** Markers of nuclear and microsomal proteins in the plasma membrane and caveolae fractions. Equal amounts of protein (10 μg) were subjected to SDS/PAGE, immunoblotting and densitometric scanning (Gel Doc 2000 imaging system, Bio-Rad). The results are expressed as the amount of the marker protein relative to the indicated subcellular fraction (set to 100%). The experiments shown are representative of 3 independent experiments. *Abbreviations*: *plasma membrane (PM) fraction; caveolae membrane/lipid raft (CM) fraction*.

	Membrane Fraction (%)			
	Plasma Membrane (PM)	Caveolae (CM)	Nuclear	Microsomal
TGN38	3.8±1.0 (<4%)	1.6±3.5 (<4%)	2.0±4.1 (<6%)	100
Nucleoporin p62	0.9±2.3 (<3%)	0.5±1.9 (<2%)	100	7.9±3.1 (<8%)

**Table 3:** Subcellular distribution of sphingomyelin in 3T3-F442A adipocytes. Cells were treated with SM-LA (15 μM) for the indicated incubation times. The results, which are expressed as μg of SM per mg protein, are presented as percentages of control cells and are the mean±SEM of three independent experiments. The SM content of plasma membranes was 30±2 μg/mg protein in control adipocytes. The caveolae fraction represents approximately one-third (0.36) of the plasma membrane [78].

	Fraction			
	PNS	PM	NCM	CM
Control	100 (%)	100 (%)	100 (%)	100 (%)
SM-LA (2 h)	110.7±6.2	121.2±9.8 *	108.6±13.4	94.4±16.7
SM-LA (4 h)	108.4±5.6	110.1±13.4	104.6±9.4	112.4±11.1
SM-LA (8 h)	110.0±2.7	116.5±11.0	109.5±13.7	105.9±15.0
SM-LA (24 h)	174.3±9.6 *	160.2±12.1 *	117.8±5.9	649.1±32.0 **
SM-LA (32 h)	182.0±11.1 *	178.6±9.5 *	106.6±7.1	598.6±41.2 *
SM-LA (48 h)	728.0±24.6 **	98.4±16.3	105.4±11.0	134.7±9.7

*P<0.05

**P<0.01; SM-treated cells compared with control cells. *Abbreviations*: *post-nuclear supernatant (PNS)*, *plasma membrane (PM) fraction*, *non-caveolae membrane/non-lipid raft (NCM) fraction*, *caveolae membrane/lipid raft (CM) fraction*. *PNS represents the fraction of the cells without nuclei including cytosol*, *microsomes*, *membranes and others*.

### Reduction in membrane fluidity because of sphingomyelin enrichment

Several studies have revealed the importance of membrane fluidity in signal transduction, and alterations in membrane fluidity may contribute to the development of diseases. To further analyze the relation between sphingomyelin and the cell membrane fluidity of 3T3-F442A adipocytes, the time response of membrane fluidity to sphingomyelin treatment was evaluated, taking into consideration that after 48 h of treatment, the SM is no longer retained in the plasma membrane and caveolae. The cells were labeled with 1,6-diphenyl-1,3,5-hexatriene (DPH), and fluorescence anisotropy of the probe was determined under conditions where DPH was primarily in the plasma membrane. The fluorescence anisotropy of DPH in untreated cells was 0.170±0.003 at 37°C and increased by 17.1% (*P*<0.005) and 4.7% (*P*<0.001) when the cells were treated with SM-LA for 24 and 48 h, respectively (Table 4). However, a significant diminution by 5.3% (*P*<0.05) was observed after 72 h of SM-LA treatment.

**Table 4:** Time-dependent response of membrane fluidity to sphingomyelin treatment. Cells were treated with SM-LA (15 μM) or vehicle for the indicated incubation times. The cells were labeled with 1,6-diphenyl-1,3,5-hexatriene (DPH), and the fluorescence anisotropy of the probe was determined. The measurements were performed at 37°C for 2 min immediately after the addition of the fluorescent probes. Each value is the mean±SEM of three independent experiments. *P*<0.05, *P*<0.005 and *P*<0.001; SM-treated cells compared with control cells.

	Fluorescence anisotropy	Percentage of Control	*P* value
Control	0.170±0.003	100	
SM-LA (2 h)	0.168±0.008	98.8	NS
SM-LA (24 h)	0.199±0.010	117.1	<0.005
SM-LA (48 h)	0.178±0.003	104.7	<0.001
SM-LA (72 h)	0.161±0.005	94.7	<0.05

A second set of experiments was next assessed (Table 5) to evaluate the effects of exogenous SMs, GSH and PPMP at 24 h of treatment. The fluorescence anisotropy of DPH in untreated cells was 0.173±0.004 and increased in SM-enriched cells by 7.5% (*P*<0.0001), 7.3% (*P*<0.0005), 12.5% (*P*<0.0001) and 9.5% (*P*<0.0001) when the cells were treated with SM-PA, SM-SNA, SM-LA or GSH, respectively. No significant changes were observed in the case of PPMP treatment (Table 5).

**Table 5:** Sphingomyelin enrichment decreased membrane fluidity in 3T3-F442A adipocytes. 3T3-F442A adipocytes were treated with 15 μM exogenous natural SM, 10 mM GSH, 20 μM PPMP or vehicle for 24 h. The cells were labeled and the measurements were performed as indicated in Table 4. Each value is the mean±SEM of four independent experiments. *P*<0.0005 and *P*<0.0001; SM-, GSH-, and PPMP-treated cells compared with untreated cells.

		Fluorescence anisotropy	Percentage of Control	*P* value
Control		0.173±0.004	100	
SM-enriched cells	SM-PA	0.186±0.006	107.5	<0.0001
SM-enriched cells	SM-SNA	0.186±0.009	107.3	<0.0005
SM-enriched cells	SM-LA	0.195±0.005	112.5	<0.0001
SM-enriched cells	N-SMase inhibitor (GSH)	0.190±0.005	109.5	<0.0001
SM-unmodulated cells	PPMP	0.171±0.010	98.8	NS

### Sphingomyelin regulated SREBPs

**Fig 4:**
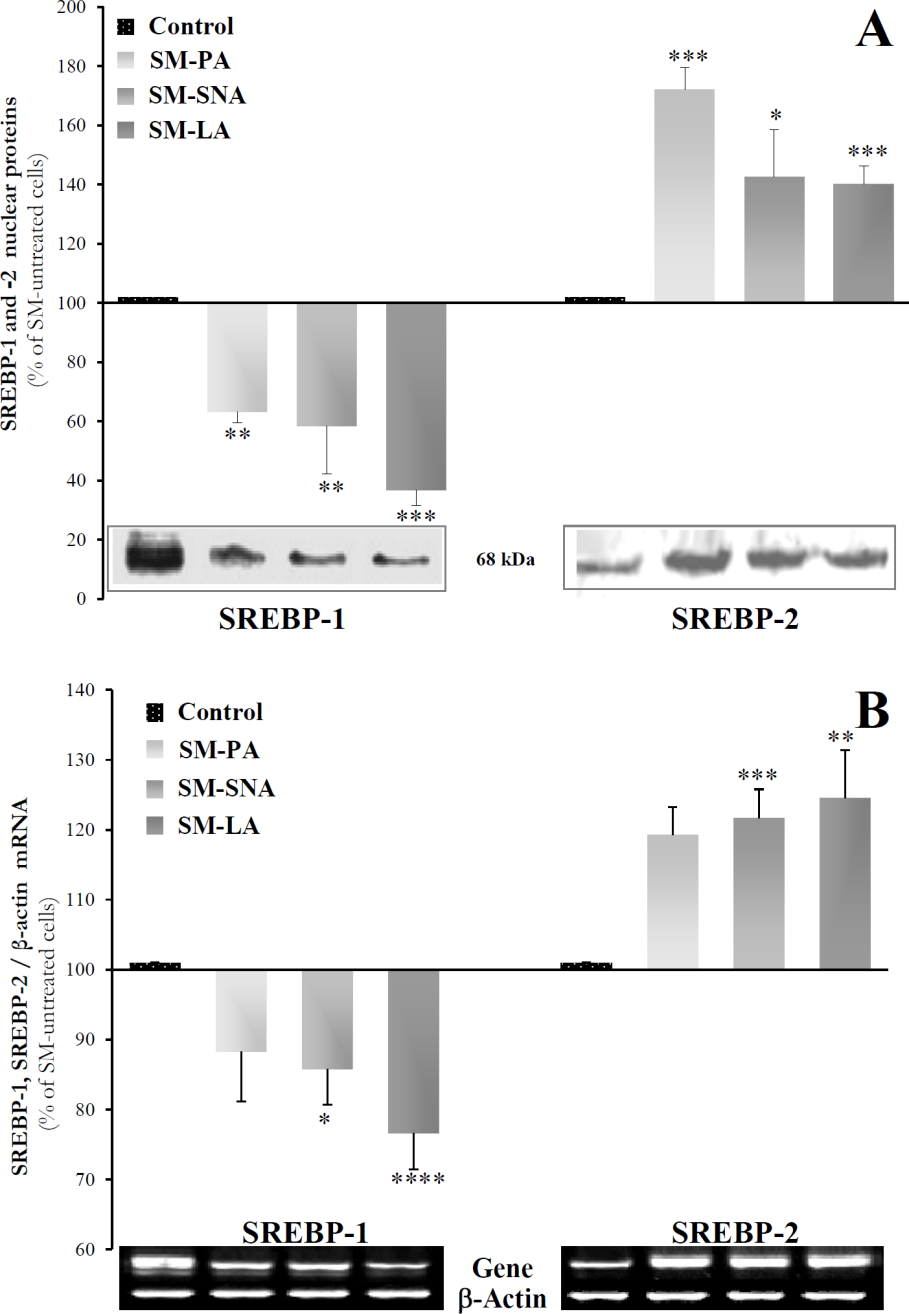
SREBP protein and mRNA expression in SM-enriched adipocytes. Cells were differentiated into adipocytes and treated with SMs as indicated in Fig 3. (A) Immunoblot analysis of nuclear SREBP proteins. Nuclear extracts were separated by SDS-PAGE (30 μg of protein) and immunoblotted with SREBP-1 (H-160) and SREBP-2 (N-19) polyclonal antibodies raised against epitopes mapping at the N-terminus of SREBP. (B) Semi-quantitative RT-PCR analysis of SREBP-1 and SREBP-2 mRNA levels. Total RNAs were reverse-transcribed and amplified by PCR as described in the Materials and methods. Amplified products were separated on an agarose gel and stained with ethidium bromide. Representative blots are shown under the histograms. The results are expressed as percentages of SM-untreated cells and are the mean±SEM of three independent experiments, performed in duplicate, triplicate or quadruplicate. ^*^ *P*<0.05, ^**^ *P*<0.01, ^***^ *P*<0.005 and ^****^ *P*<0.001; SM-treated cells compared with untreated cells.

First, the expression of SREBPs proteins was examined in SM-enriched adipocytes (Fig 4A and 4B). The nuclear fraction was immunoblotted with antibodies that recognize the active N-terminus of SREBP proteins. A 68 kDa band was detected in the nuclear fraction and decreased in the case of SREBP-1 [-37% (*P*<0.01), -42% (*P*<0.01), -63% (*P*<0.005)] but increased in the case of SREBP-2 [71% (*P*<0.005), 42% (*P*<0.01), 40% (*P*<0.005)] by the SM treatments (Fig 4A). The down-regulation of SREBP-1 and the up-regulation of SREBP-2 proteins led us to investigate whether the transcriptional levels could be affected in SM-enriched adipocytes. First, the mRNA levels were estimated by conventional semi-quantitative RT-PCR (Fig 4B). β-Actin was selected as the housekeeping gene due to its stable expression across the treatments. The SREBP-1 mRNA levels were reduced by -12% (NS), -14% (*P*<0.05) and -23% (*P*<0.001), whereas SREBP-2 mRNAs increased by 19% (NS), 22% (*P*<0.005) and 25% (*P*<0.01) with SM-PA, SM-SNA and SM-LA treatments, respectively. Additionally, the semi-quantitative PCR results were validated by real-time PCR, and the effects of GSH and PPMP were also investigated. The melting curves of the three genes revealed the absence of non-specific products. SM, GSH or PPMP treatments did not induce any significant change in the amounts of β-actin mRNA. The data indicated that the regulation of SREBPs in adipocytes treated by N-SMase inhibitor or exogenous SM followed identical patterns. In cells treated with exogenous SMs, SREBP transcript expression significantly decreased by -2.5-fold for SREBP-1 (*P*<0.0001) but increased by 2.44-fold for SREBP-2 (*P*<0.0001) (Table 6). However, the changes in SREBP-1 were more noticeable after GSH treatment; SREBP-1 gene expression decreased by approximately 5-fold. Interestingly, the changes were moderate in SM-unmodulated cells treated with PPMP. However, these changes were opposite those changes observed for the SM-enriched cells (Table 6); we noted a significant increase in SREBP-1 expression. To examine whether the changes in mRNA levels were due to the modulation of gene transcription or to changes in mRNA stability, we used the transcription inhibitor actinomycin D. 3T3-F442A adipocytes were treated with SM for 24 h, as described previously, followed by treatment with actinomycin D (5 μg/ml). Total RNAs were subsequently extracted at time points between 0 and 8 h. The mRNA levels of SREBP-1 and SREBP-2 were determined as previously described and normalized to β-actin. Regardless of the studied gene, the degradation rate of mRNAs in SM-enriched cells did not significantly differ from that in control cells, suggesting that SM did not affect mRNA stability but regulated gene expression at the transcriptional level (data not shown).

**Table 6:** Real-time quantitative RT-PCR determination of SREBP-1 and SREBP-2 mRNA levels in SM-enriched adipocytes or unmodulated adipocytes. 3T3-F442A adipocytes were treated with 15 μM natural SM (SM-LA, 24 h), 10 mM GSH (24 h), 20 μM PPMP (24 h) or vehicle. The mRNA levels of the studied genes were determined. The results are expressed as-fold variations over respective controls (R) after normalization to β-actin, as indicated in the Materials and methods. R values superior or equal to 2 were considered positive regulation of gene expression, whereas values lower than 0.5 indicated negative regulation. The results presented are the means of 3 independent experiments, which were performed twice each in duplicate.

		SREBP-1		SREBP-2	
		R	*P value*	R	*P value*
Control		1^a^		1	
SM-enriched cells	SM-LA	-2.50	<0.0001	2.44	<0.0001
SM-enriched cells	N-SMase inhibitor (GSH)	-5.42	<0.0001	2.82	<0.001
SM-unmodulated cells	PPMP	1.99	<0.05	1 (0.65)	<0.01

^a^ The control group was expressed as 1. *P*<0.0001, *P*<0.001, *P*<0.01 and *P*<0.05; SM-, GSH-, and PPMP-treated cells compared with untreated cells.

### SM-enrichment inhibited ERK but not p38 or JNK

**Fig 5:**
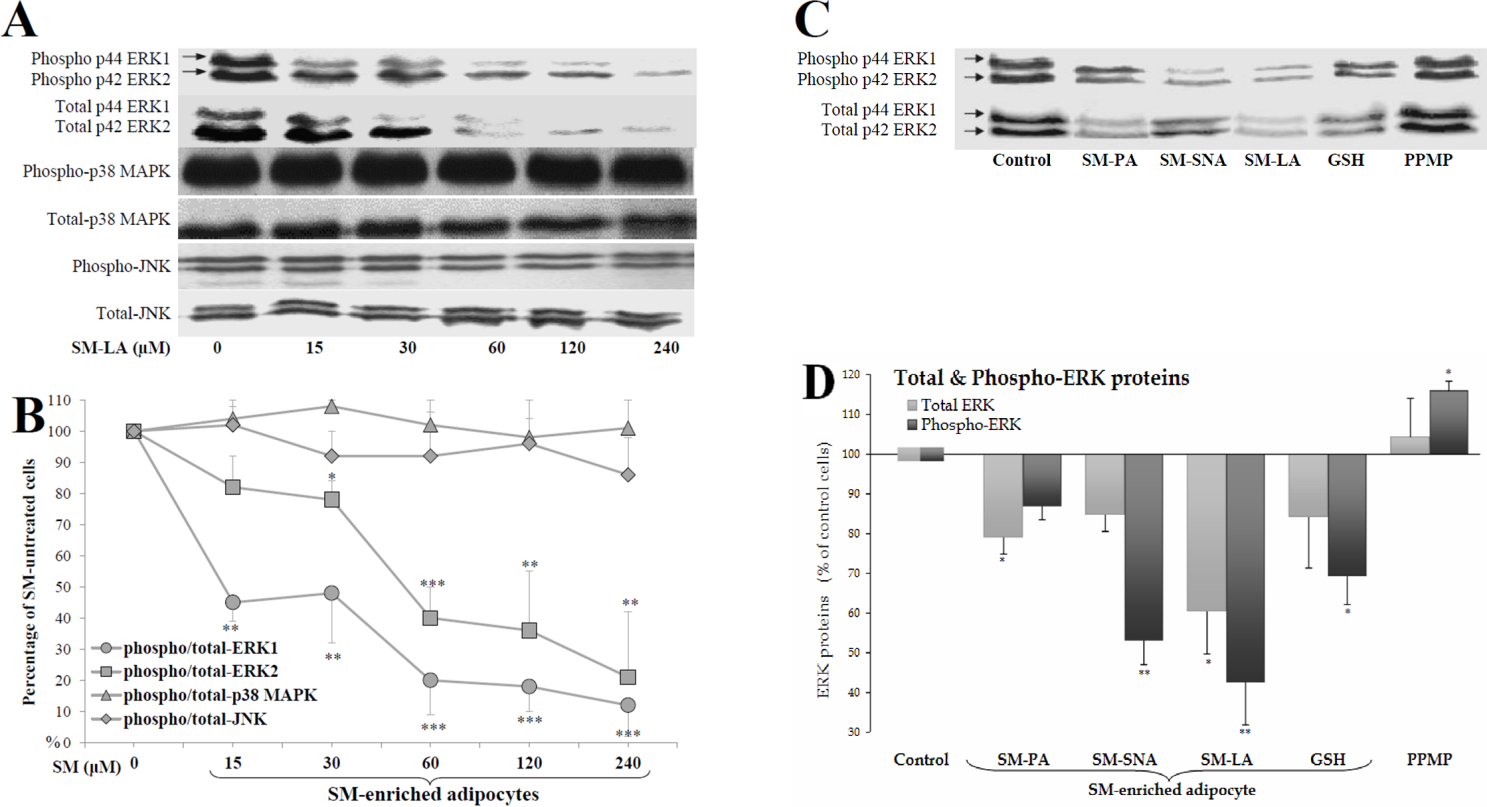
Inhibition of ERK proteins, but not p38 MAPK and JNK, in SM-enriched adipocytes. Adipocytes were treated for 24 h with the indicated concentrations of SM-LA (A and B) or 30 μM SM, 10 mM GSH, 20 μM PPMP or vehicle (C and D). Cell lysates (40 μg of proteins) were separated by SDS-PAGE and immunoblotted using antibodies recognizing (1) the phosphorylated (activated) forms of ERK (p44/42 Tyr204), p38 (Tyr180/Tyr182), JNK (Thr183/ Tyr185), and (2) total ERK, p38, and JNK proteins. (A,C) Representative blots of phosphorylated and total proteins are shown. (B) Quantitative variations in phospho-ERK/JNK/p38 amounts relative to total proteins. (D) Quantitative variations in total and phospho-ERK amounts: the blots of p42 and p44 ERKs shown in (C) were combined to quantify the amounts of total ERK1/2 proteins. The results are expressed as percentages of control cells and are the mean±SEM of four independent experiments, which were each performed in duplicate. ^*^ *P*<0.05, ^**^ *P*<0.01 and ^***^ *P*<0.005; SM-, GSH-, and PPMP-treated cells compared with untreated cells.

MAP kinase is a family of evolutionarily conserved serine/threonine kinases that includes ERK1/2, SAPK/JNK, and p38. In support of the strong linkage between the MAP kinase-dependent pathway and SREBP-1 expression and to better understand the physiological implications of SM actions, we determined whether the phosphorylation of p44/42 ERK, JNK and p38 MAP kinases was affected by SM. We found that SM promoted the inhibition of ERK1/2 phosphorylation in a dose-dependent manner but had no effects on p38 or JNK (Fig 5A and 5B). SM caused the inhibition of the basal levels of phospho-ERK1 and phospho-ERK2 proteins in a dose-dependent manner, with 50% effective doses (ED_50_) of approximately 30 μM and 40 μM, respectively.

Then, we measured the amounts of total and phosphorylated ERK proteins in cells incubated with or without natural SM, GSH and PPMP compounds. As shown in Fig 5C, the levels of phospho-ERK were reduced in SM-enriched cells parallel to a decrease in the total levels of the protein. The decreased levels of phospho-ERK1/2 were -13% (NS), -47% (*P*<0.005), -57% (*P*<0.01) and -31% (*P*<0.05) when the cells were treated with SM-PA, SM-SNA, SM-LA and GSH, respectively (Fig 5D). In contrast, the amount of phosphorylated ERK proteins increased in PPMP-treated cells (16%, *P*<0.05).

### ERK phosphorylation was required for SREBP-1 inhibition in SM-enriched cells

**Fig 6:**
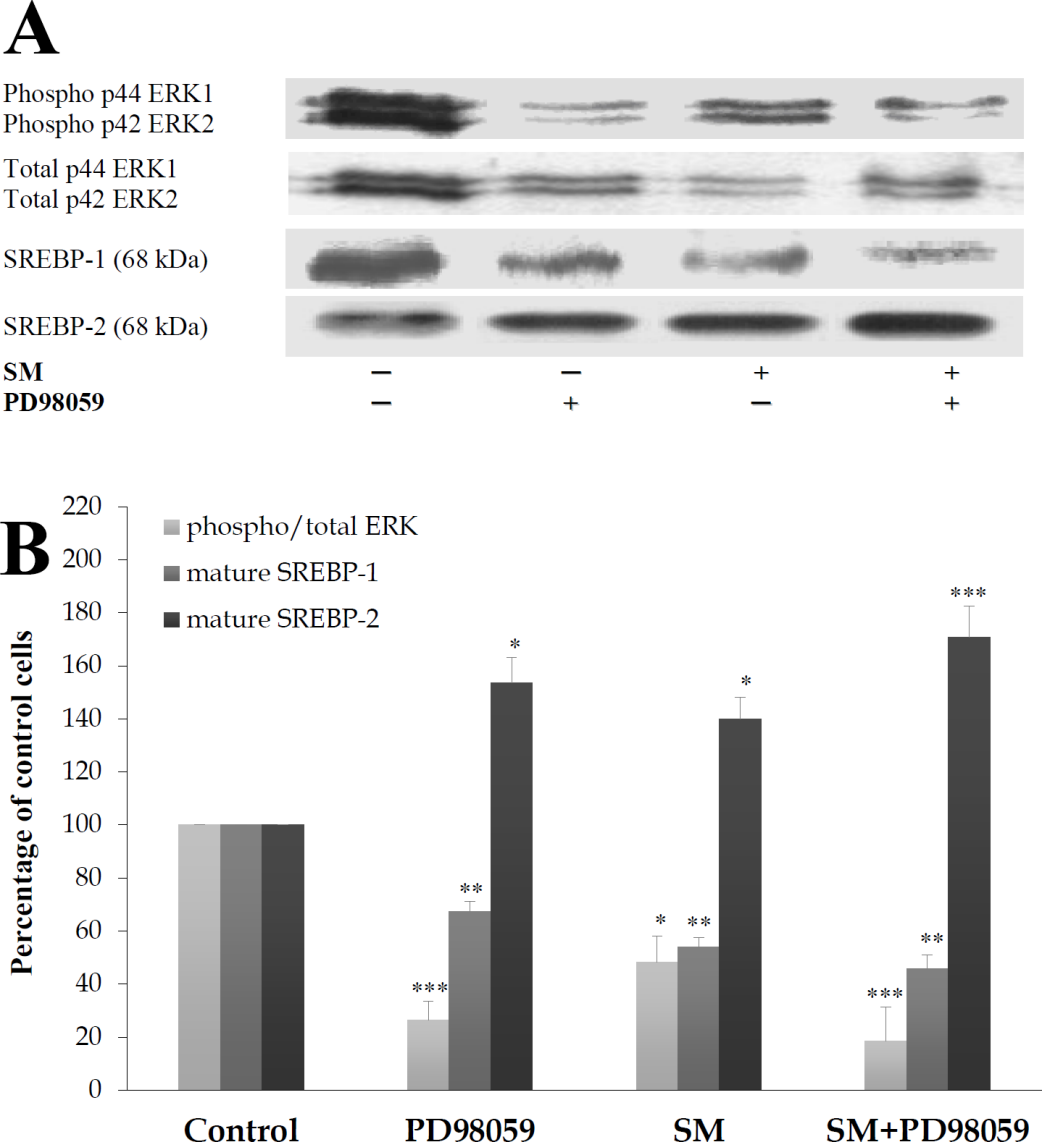
MEK1/2 selective inhibitor (PD98059) and SM reduce the phosphorylation of ERK and SREBP-1. Cells were treated 24 h with 50 μM PD98059 with or without 30 μM SM-LA. Cell lysates were separated by SDS-PAGE and immunoblotted with affinity-purified: (1) monoclonal antibody raised against a sequence containing phosphorylated Tyr204 of ERK1/2 and polyclonal antibody raised against a peptide mapping subdomain X1 of ERK and (2) with polyclonal antibodies raised against SREBP-1 or SREBP-2, as indicated in Fig 4. Representative blots (A) and quantitative variations (B) are shown. The results are expressed as the percentages of maximum and are the mean±SEM of four (ERK and SREBP-1) and six (SREBP-2) independent experiments, which were each performed in duplicate. ^*^ *P*<0.05, ^**^ *P*<0.01 and ^***^ *P*<0.005; SM/ PD98059-treated cells compared with control cells.

To determine whether ERK is required for the SM action, the role of PD98059, which is a selective inhibitor of MEK1/2 (the direct upstream kinase activator of ERK1/2) that inhibits ERK1/2 phosphorylation, was examined (Fig 6). PD98059 strongly inhibited the phosphorylation of ERK1/2 proteins (-71%, *P*<0.005) and significantly diminished the levels of the p68 SREBP-1 proteins (-34%, *P*<0.01) in SM-untreated cells. In SM-treated cells, the reduction in ERK phosphorylation by PD98059 was maintained (-84%, *P*<0.005), and the reduction in the expression of the mature SREBP-1 protein was reinforced (-55%, *P*<0.01). PD98059 is known to inhibit SREBP-1 expression; however, SREBP-2 seems to be up-regulated by PD98059 in control and SM-enriched 3T3-F442A cells, with expression levels of 54% (*P* = 0.031), 40% (*P* = 0.015) and 72% (*P =* 0.003) when these cells were treated with PD98059, SM and SM+PD98059, respectively.

### p-ERK inhibition required Ras, Raf and MEK in SM-enriched cells

**Fig 7:**
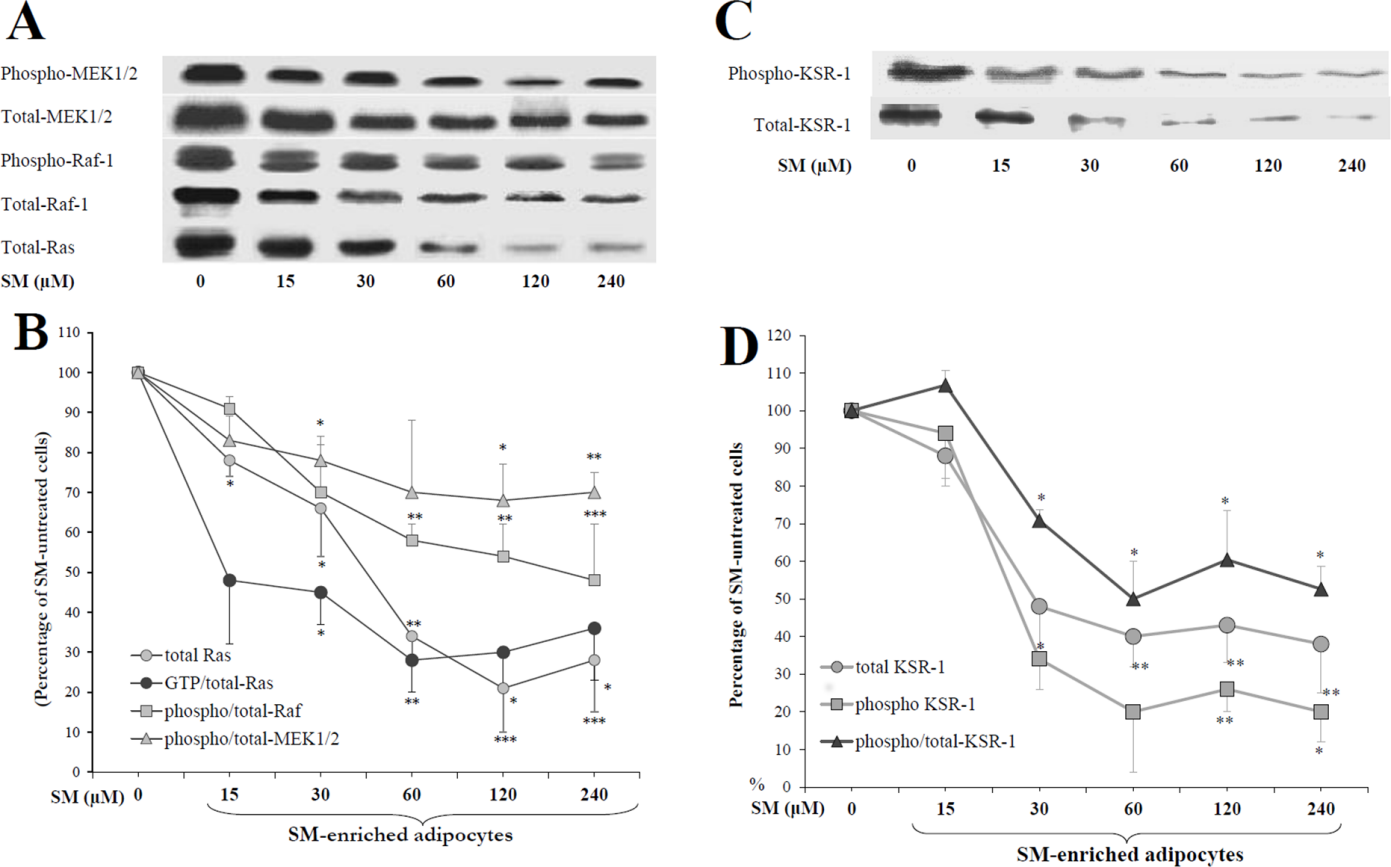
Inhibition of Ras, Raf, MEK(A and B)and KSR (C and D) by SM in 3T3-F442A adipocytes. Cells were treated for 24 h with the indicated concentrations of SM-LA. Cell lysates (30–60 μg of protein) were separated by SDS-PAGE and immunoblotted with antibodies recognizing (1) the phosphorylated (activated) forms of Raf-1 (Tyr340/341), MEK-1/2 (Ser218/222) and KSR-1 (Ser392) and (2) total pan-Ras (N/H/K-Ras), Raf-1, MEK1/2 and KSR-1. Quantitative variations in phosphorytated amounts relative to total proteins are shown. The Ras activity was determined by ELISA (60 μg of cell lysate protein): active GTP-bound state of Ras is detected and measured quantitatively through the addition of a monoclonal anti-Ras antibody that detects K-, H-, N- Ras isoforms. GTP-Ras is normalized to the total-Ras. The results are expressed as percentages of SM-untreated cells and are the mean±SEM of four independent experiments, which were each performed twice. ^*^ *P*<0.05, ^**^ *P*<0.01 and ^***^ *P*<0.005; SM-treated cells compared with untreated cells.

To elucidate whether Ras/Raf/MEK are required for ERK inhibition by membrane SMs, cells were treated with various concentrations of SM-LA (0–240 μM), and anti-phosphoMEK1/2, anti-phosphoRaf-1 and anti-Ras immunoprecipitates were analyzed (Fig 7A and 7B). SM reduced the levels of phosphorylated proteins as well as the total proteins in a dose-dependent manner. Remarkably, SM dramatically reduced Ras protein levels; the maximal inhibition reached -80% (*P*<0.005) with 120 μM of SM. For the same concentration of SM, the ratio of phospho/total-Raf-1 and phospho/total-MEK1/2 protein levels decreased by -44% (*P*<0.005) and -33% (*P*<0.01), respectively.

Ras proteins function as GDP/GTP-regulated binary switches in signal transduction cascades. Ras activity was determined by ELISA by detecting the active Ras (GTP bound state) relative to the total-Ras proteins. We observed a significant decrease in the activation of Ras by SM-LA (0–240 μM), reaching a maximal inhibition value of -72% (*P*<0.01) with 60 μM of SM (Fig 7B). These decreases are consistent with our findings for ERK1/2 regulation by SM.

### Sphingomyelin down-regulated the kinase suppressor of Ras (KSR-1)

The next set of experiments examined the effects of SM on KSR, the Kinase Suppressor of Ras. KSR is a molecular scaffold that interacts with the components of the Raf/MEK/ERK kinase cascade and that positively regulates ERK signaling. KSR was defined in genetic screens as downstream of Ras and either upstream or parallel to Raf-1 [42]. KSR facilitates the phosphorylation of MEK by RAF. In mammalian cells, ceramide-activated protein kinase (CAPK) has been considered the counterpart of KSR [43], implicating Raf-1 as a ceramide-activated kinase. Cells were treated with increasing concentrations of SM-LA for 24 h. Whole-cell extracts were immunoblotted using a KSR-1 and phospho-KSR-1 antibodies. SM treatment caused decreases in the phosphorylated and total levels of KSR-1 protein in a dose-dependent manner (Fig 7C and 7D).

### SM-enriched cells expressed elevated levels of caveolins

Another distinctive characteristic of the SM-enriched cells could be altered expression of the caveolin gene. Based on several important observations, one of the most attractive candidates that links the Ras-p42/44 MAP kinase signaling to SREBP is caveolin [19,20]. Caveolins are cholesterol-binding integral membrane proteins that are distributed primarily at the plasma membrane but also in the Golgi complex. These proteins exist as three distinct isoforms, where caveolin 1 (Cav-1) and 2 (Cav-2) are strictly co-localized within the plasma membrane [44]. Therefore, we were interested in elucidating any possible relation between Cav-1 and Cav-2 and SM. First, cells were treated for 24 h with SM-LA (30 μM), GSH (10 mM), PPMP (20 μM) or vehicle, and the expression levels of Cav-1 and Cav-2 mRNA were examined. Cav-1 and Cav-2 mRNA were enhanced in the presence of exogenous SM-LA (4.01 and 2.35 fold) or GSH (5.37 and 4.64 fold), respectively (Table 7), whereas no changes were observed in PPMP-treated cells. Caveolins mRNA stability were also assessed, as previously described, using the transcription inhibitor actinomycin D. The degradation rate in SM-enriched cells did not significantly differ from that in control cells, suggesting that SM regulates gene expression at the transcriptional level (data not shown).

**Table 7:** Real-time RT-PCR determination of caveolins mRNA levels in SM-enriched adipocytes or unmodulated adipocytes. 3T3-F442A adipocytes were treated with 30 μM exogenous natural SM (SM-LA, 24 h), 10 mM GSH (24 h), 20 μM PPMP (24 h) or vehicle. The results are expressed as-fold variation over respective controls (R) after normalization to β-actin, as indicated in the Materials and methods. R values greater than or equal to 2 were considered positive regulation of gene expression, whereas values lower than 0.5 indicated negative regulation. The results presented are the means of 3 independent experiments, which were performed twice each in duplicate.

		Cav-1		Cav-2	
		R	*P value*	R	*P value*
Control		1^a^		1^a^	
SM-enriched cells	SM-LA	4.01	<0.005	2.35	<0.0001
SM-enriched cells	N-SMase inhibitor (GSH)	5.37	<0.01	4.64	<0.0001
SM-unmodulated cells	PPMP	1 (1.19)	<0.001	1 (0.86)	<0.05

^a^ The control group was expressed as 1. *P*<0.0001 and *P*<0.05; SM-, GSH-, and PPMP-treated cells compared with untreated cells.

**Fig 8:**
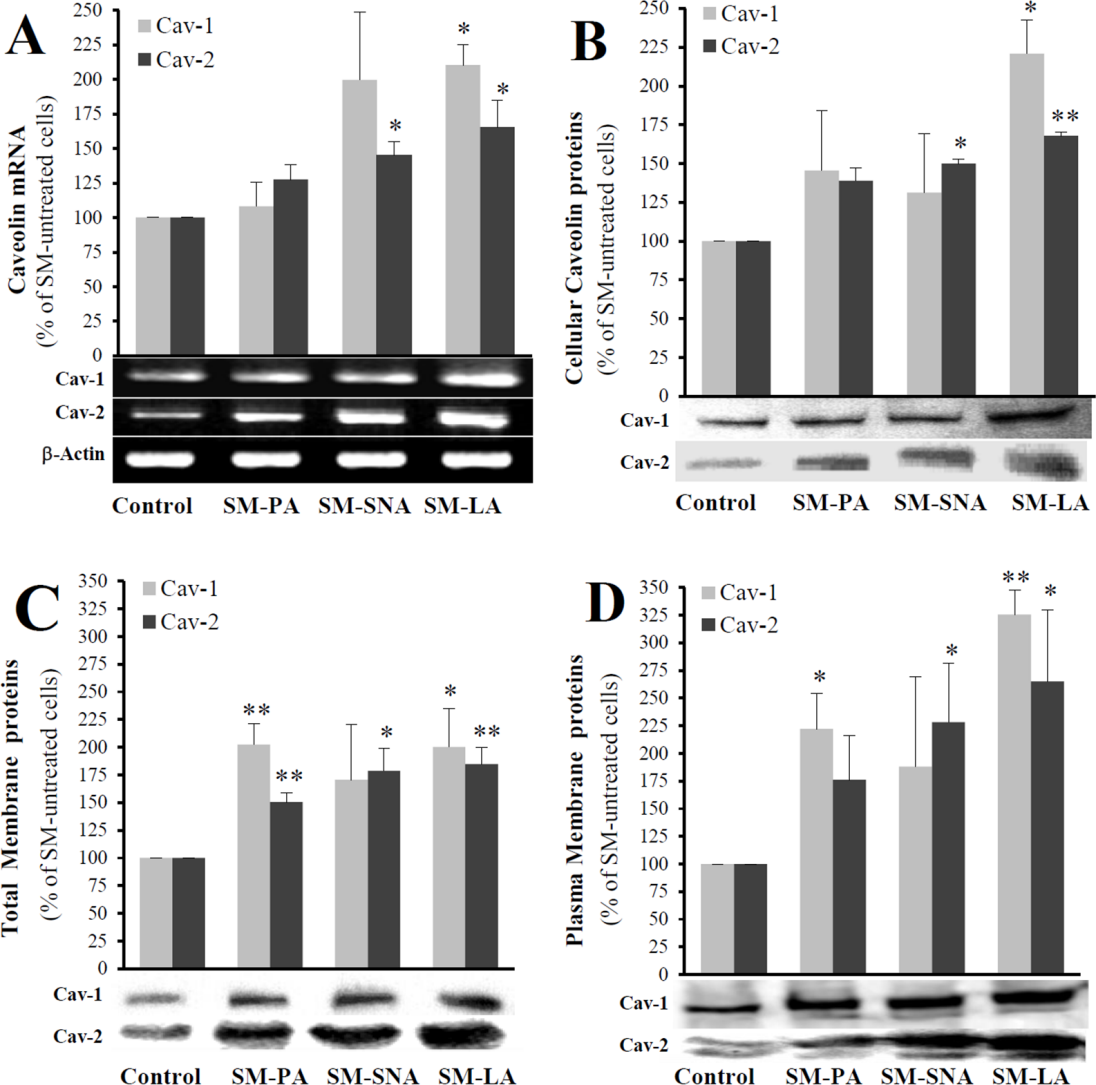
Effects of exogenous sphingomyelins on caveolin expression. Cells treated with SMs as indicated in Fig 3. (A) Total RNAs were reverse-transcribed and amplified by PCR as described in the materials and methods. (B, C, and D) Lysates were separated by SDS-PAGE and immunoblotted with (1) a polyclonal antibody (N-20) and (2) a monoclonal antibody (IgG1, clone 65) that recognize Cav-1 and Cav-2, respectively (25 μg of proteins). A representative western blot is shown under each series of histograms, representing the expression levels of caveolins proteins: cellular (B), total membrane (C) and plasma membrane (D). The results are expressed as percentages of SM-untreated cells and are the mean±SEM of three independent experiments, which were performed in duplicate for mRNA and in triplicate for proteins. ^*^ *P*<0.05 and ^**^ *P*<0.01; SM-treated cells compared with untreated cells.

Caveolins were significantly more abundant in the case of SM-LA treatment (Fig 8). The increases for Cav-1 and Cav-2 were respectively 2.1- and 1.7-fold for mRNA, and 2.2- and 1.7-fold for the cellular protein. In total membranes, the increases were: 2.0- and 1.8-fold respectively. Interestingly, the amounts of caveolins proteins were markedly higher (3.3- and 2.7-fold, respectively) in the plasma membranes of SM-enriched adipocytes.

### SM-enriched cells accumulated free cholesterol

**Fig 9:**
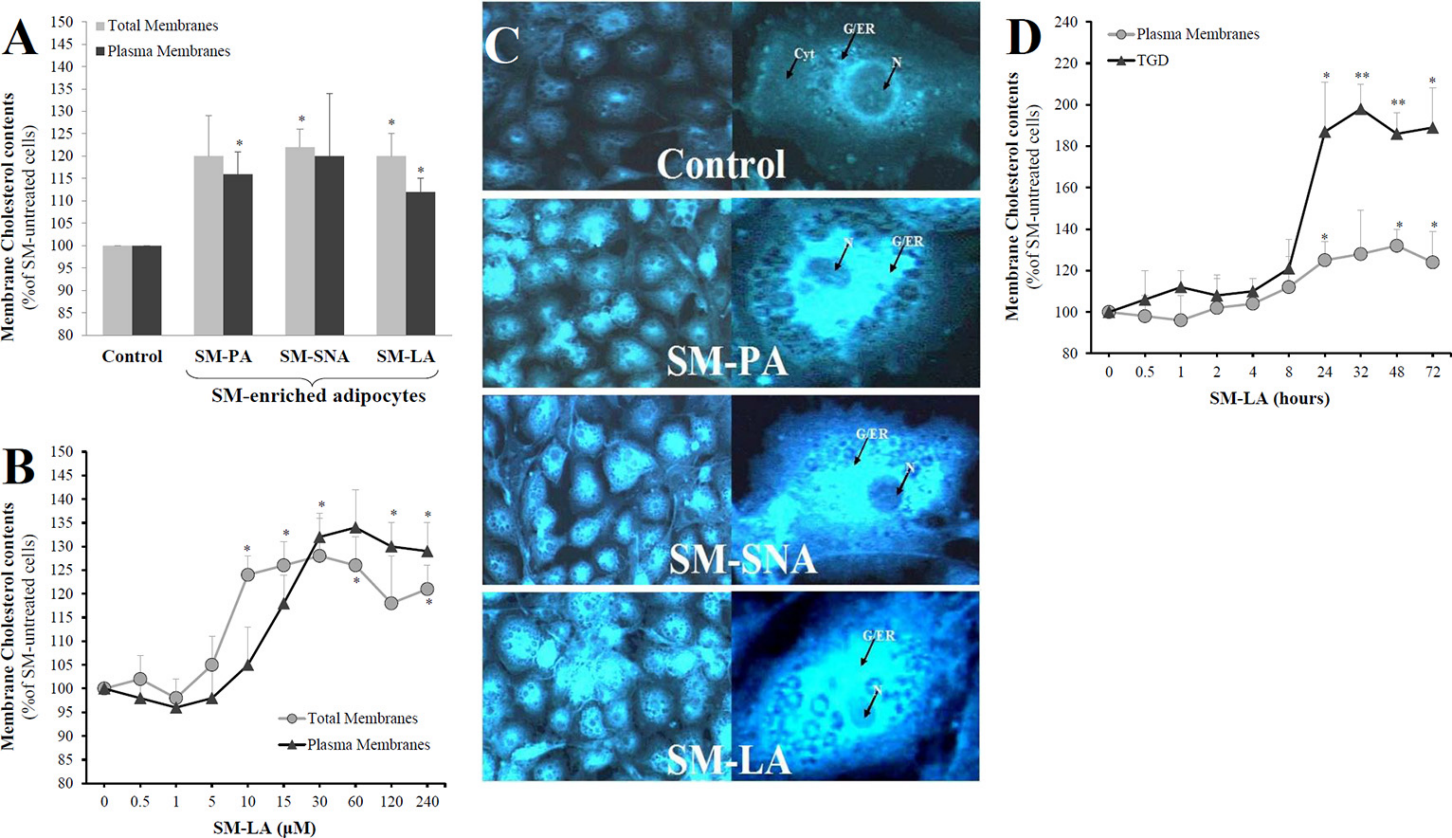
SMs modulate free cholesterol (CHOL) distribution in 3T3-F442A adipocytes. Total and plasma membranes, and cytosol were isolated from differentiated adipocytes and the level of CHOL was determined as indicated in methods. (A) Cells were treated for 24 h with SMs as indicated in Fig 3. (B) Cells were treated with SM-LA (24 h) with the indicated concentrations. (C) Mature adipocytes were fixed, stained with 100 μg/ml filipin for 3 h in the dark and visualized by fluorescence microscopy. From the top to the bottom of each column, representative examples of control cells, SM-PA-, SM-SNA- and SM-LA-treated cells are shown. One single representative adipocyte is shown on the right in each case. Cyt: cytoplasm; N: nucleus; G/ER: Golgi system and endoplasmic reticulum. (D) The distribution of CHOL between plasma membranes and triglyceride droplets (TGD) was examined by treating the cells with SM-LA (30 μM) for the indicated time. The results are expressed as μg of CHOL per mg protein and as percentages of SM-untreated cells and are the mean±SEM of four (A,B) or three (C,D) independent experiments, which were performed in triplicate. ^*^ *P* < 0.05 and ^**^ *P*<0.01; SM-treated cells compared with untreated cells.

The up-regulation of SREBP-2 and caveolin by SMs could affect the homeostasis of CHOL and/or its intracellular distribution. First, cells were treated with exogenous SMs (15 μM) for 24 h; no published work had treated cells with different exogenous SMs (SM-PA, SM-SNA, and SM-LA in the present study) and studied their effects on cholesterol. The quantitative determination of CHOL content in total and plasma membranes (Fig 9A) indicated that the CHOL content increased by approximately 13–22% in SM-enriched adipocytes. No marked differences were observed among the three used SMs. Next, elevated concentrations of SM-LA (up to 240 μM) were examined to determine whether the absence of effects were due to insignificant concentrations of SM. Cholesterol increased in the total and plasma membrane but the increases were also moderate (18–32%) when cells were treated with SM-LA with concentrations superior to 10 μM (Fig 9B). To determine whether the absence of variations of CHOL content between SM-PA, SM-SNA and SM-LA treatments was due to the sequestration of CHOL away from the membrane, the adipocytes were stained with filipin, which is a fluorescent free-CHOL-binding molecule. The intensity of filipin staining was moderate in control cells. In contrast, SM-enriched cells displayed bright fluorescence in the entire cell, thus indicating and confirming the accumulation of cellular CHOL (Fig 9C). Hypothesizing that CHOL might be sequestered in intracellular compartments, in particular the triglyceride droplets (TGD), the distribution of CHOL between plasma membranes and TGD was then examined by treating the cells with SM-LA (30 μM) for the indicated time in Fig 9D; the CHOL accumulated significantly in TGD (1.86 to 1.98 fold of increases) after 24 h of SM treatments. However, the levels of CHOL are maintained in the plasma membrane (maximum increase was 1.32 fold).

### GW4869, the N-SMase selective inhibitor, inhibited SREBP-1 and the phosphorylation of ERK proteins

**Fig 10:**
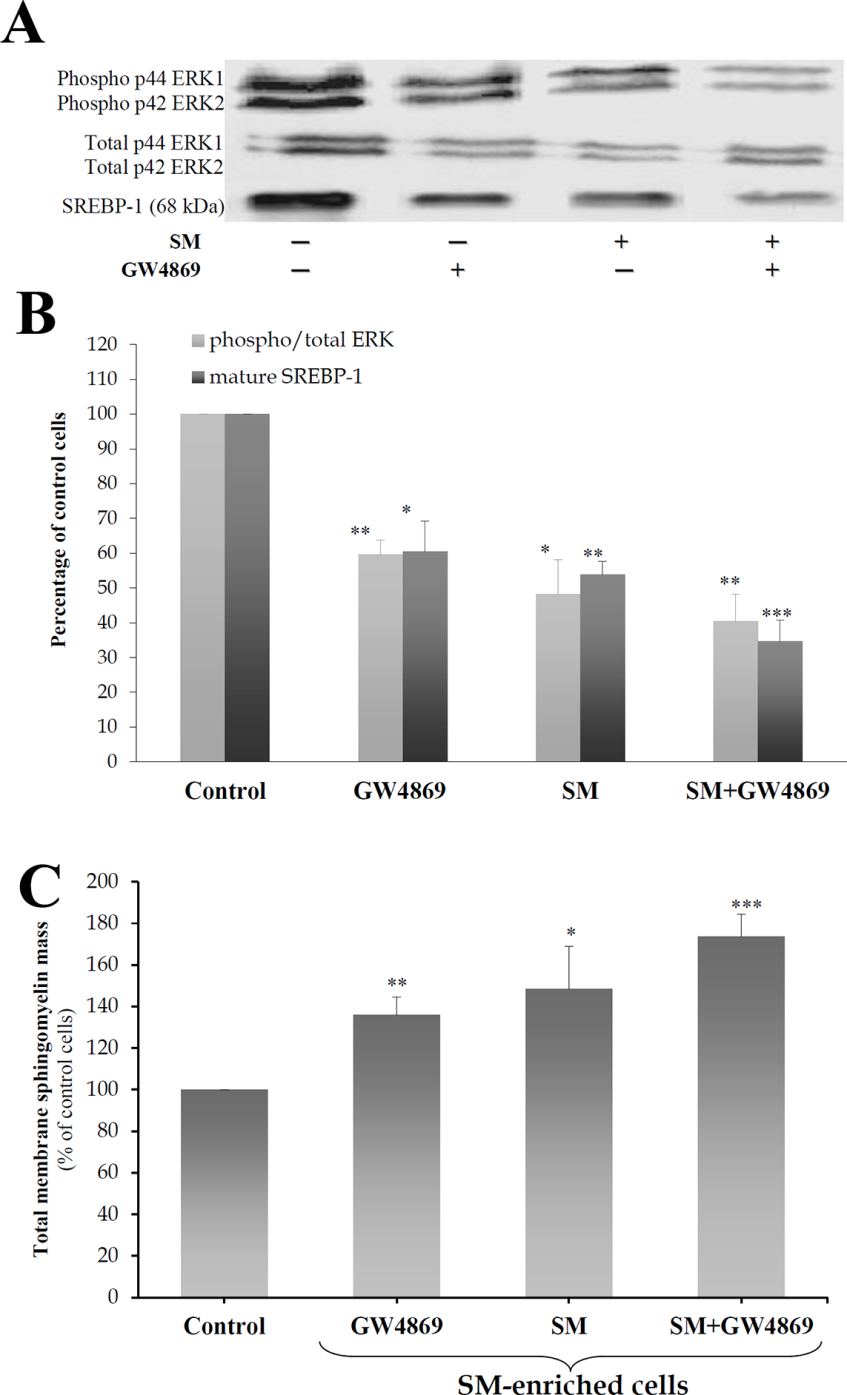
Neutral sphingomyelinase-selective inhibitor GW4869 inhibits the phosphorylation of ERK and SREBP-1 proteins. Cells were treated for 24 h with 20 μM GW4869 with or without 30 μM SM-LA. Cell lysates were separated by SDS-PAGE and immunoblotted with an affinity-purified (1) monoclonal antibody raised against a sequence containing phosphorylated Tyr204 of ERK1/2 and polyclonal antibody raised against a peptide mapping subdomain X1 of ERK (40 μg of protein) and (2) polyclonal antibody raised against epitope mapping at the N-terminus of SREBP-1 (80 μg of protein). Representative blots (A) and quantitative variations (B) are shown. The results are expressed as percentages of maximum and are the mean±SEM of four independent experiments. (C) Enrichment of the SM content in total membranes of 3T3-F442A adipocytes. Total membranes were prepared, and SM concentrations were determined as described in the Materials and methods. The results are expressed as μg of SM per mg protein, are presented as percentages of control cells, and are the mean±SEM of three independent experiments. ^*^ *P*<0.05, ^**^ *P*<0.01 and ^***^ *P*<0.005; SM- and GW4869-treated cells compared with control cells.

To confirm the absence of ceramide effects in response to SM, N-SMase activity was inhibited by treating 3T3-F442A adipocytes with the selective inhibitor of N-SMase GW4869 [45] (20 μM, 24 h), with or without exogenous SM-LA (30 μM, 24 h). GW4869 reduced both phospho/total-ERK ratio (-42%, *P*<0.01) and SREBP-1 (-39%, *P*<0.05) proteins (Fig 10A and 10B). When combined with SM-LA, the inhibition levels were -60% (*P*<0.05) for phospho-ERK and -66% (*P*<0.005) for p68 SREBP-1 proteins. The inhibition of N-SMase by GW4869 induced a significant accumulation of SM in membranes (36%, *P*<0.01); however, when the cells were treated with SM+GW4869, the increased accumulation was larger (74%, *P*<0.005) (Fig 10C). These results suggest that the action of SM was not mediated by ceramide but occurred upstream of the ceramide step instead.

### Ceramide accumulated in SM-modulated and unmodulated cells, in contrast to glucosylceramide, which only accumulated in SM-enriched cells

**Fig 11:**
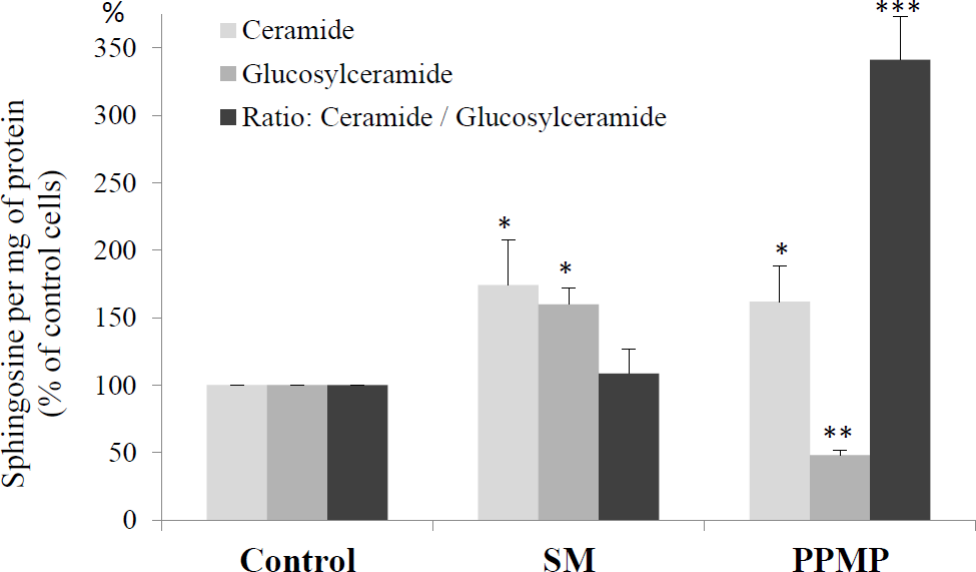
SM-enriched adipocytes accumulate glucosylceramide, unlike SM-unmodulated cells. Cells were treated for 24 h with 30 μM SM-LA, 20 μM PPMP or vehicle. After lipid isolation, ceramide and glucosylceramide were quantified as described in the Materials and methods. The results are expressed in pmol of sphingosine per mg of protein. The results presented are the means of 3 independent experiments, which were performed in triplicate. The control group was expressed as 100, and the results are shown as percentages of the control cells. ^***^ *P*<0.005, ^**^ *P*<0.01 and ^*^ *P*<0.05; SM-, GSH-, and PPMP-treated cells compared with control cells.

The next set of experiments was performed to determine the concentrations of ceramide in control and SM-treated cells. Ceramide, glucosylceramide and galactosylceramide concentrations were measured in (1) SM-enriched cells (treated 24 h with 30 μM SM-LA) and (2) SM-unmodulated cells (treated with 20 μM PPMP) and (3) controls (cells treated with vehicle). Endogenous galactosylceramide was undetectable, unlike glucosylceramide and total ceramide. In control adipocytes, the levels of ceramide and glucosylceramide were 0.116 and 0.025 pmol of sphingosine/mg protein, respectively. We observed accumulations of ceramide in both cases (+74% (*P*<0.05) and +62% (*P*<0.05) for both SM- and PPMP-treated cells, respectively). However, glucosylceramide accumulated only in the case of SM-treated cells (+40%, *P*<0.05), unlike the PPMP-treated cells, where glucosylceramide levels decreased (-48%, *P*<0.01) (Fig 11).

### Rosiglitazone reversed the effects of SM on SREBP-1, PPARγ and p-CREB

**Fig 12:**
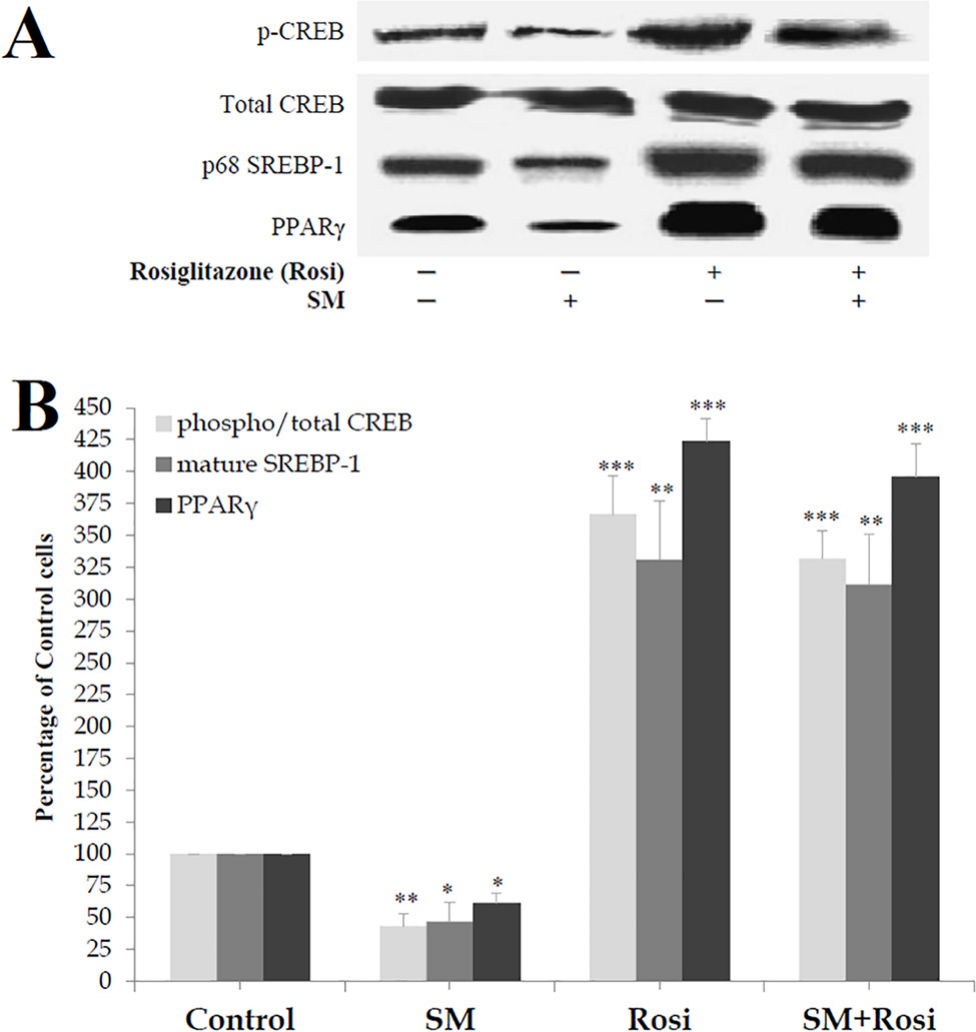
Rosiglitazone reverses the effects of SM in 3T3-F442A adipocytes. Cells were treated for 24 h with 6 μM rosiglitazone (Rosi) with or without 30 μM SM-LA. Cell lysates (80 μg of protein) were separated by SDS-PAGE and immunoblotted with an affinity-purified polyclonal antibody raised against (1) a peptide mapping within the alpha region of CREB-1 p43, (2) a short amino acid sequence containing phosphorylated Ser 133 of CREB-1, (3) amino acids 8–106 of PPARγ and (4) epitope mapping at the N-terminus of SREBP-1. Representative blots (A) and quantitative variations (B) are shown. The results are expressed as percentages of control cells and are the mean±SEM of four independent experiments, which were each performed in duplicate. ^*^ *P*<0.05, ^**^ *P*<0.01 and ^***^ *P*<0.005; SM-, Rosi-, and SM+Rosi-treated cells compared with control cells.

To verify whether the effects of SM could be reversed, SM-pretreated cells (SM-LA 24 h, 30 μM) were treated with the anti-diabetic agent rosiglitazone (24 h incubation, 6 μM), which is a potent activator of PPARγ [46,47] (Fig 12). In addition to SREBP-1 expression, the expression of two other factors downstream and upstream of SREBP-1 activation, PPARγ and CREB (cAMP-responsive element binding protein), respectively, was evaluated. Based on several important lines of evidence, PPARγ is considered a positively regulated SREBP-1 target gene [48], and CREB is considered an upstream co-regulator of SREBP-1 [49–52].

First, SM-enriched cells expressed low levels of PPARγ (-39%, *P*<0.05), mature SREBP-1 (-53%, *P*<0.05) and phospho-CREB (-59%, *P*<0.01) proteins without significant variations in total CREB protein levels (Fig 12). As expected, rosiglitazone strongly induced the expression of the studied proteins PPARγ (324%, *P*<0.005), mature SREBP-1 (231%, *P*<0.01) and phospho/total-CREB (267%, *P*<0.005) in control SM-untreated cells. Rosiglitazone significantly reversed the decrease in the phosphorylation of CREB (232%, *P*<0.005) proteins, as well as the expression of PPARγ (296%, *P*<0.005) and SREBP-1 (211%, *P*<0.01) proteins, in SM-enriched cells, thus indicating that the antidiabetic agent rosiglitazone may be considered an anti-SM in adipocytes, promoting the reversibility of SM effects.

### SM inhibited insulin-stimulated expression of SREBP-1 and PPARγ

**Fig 13:**
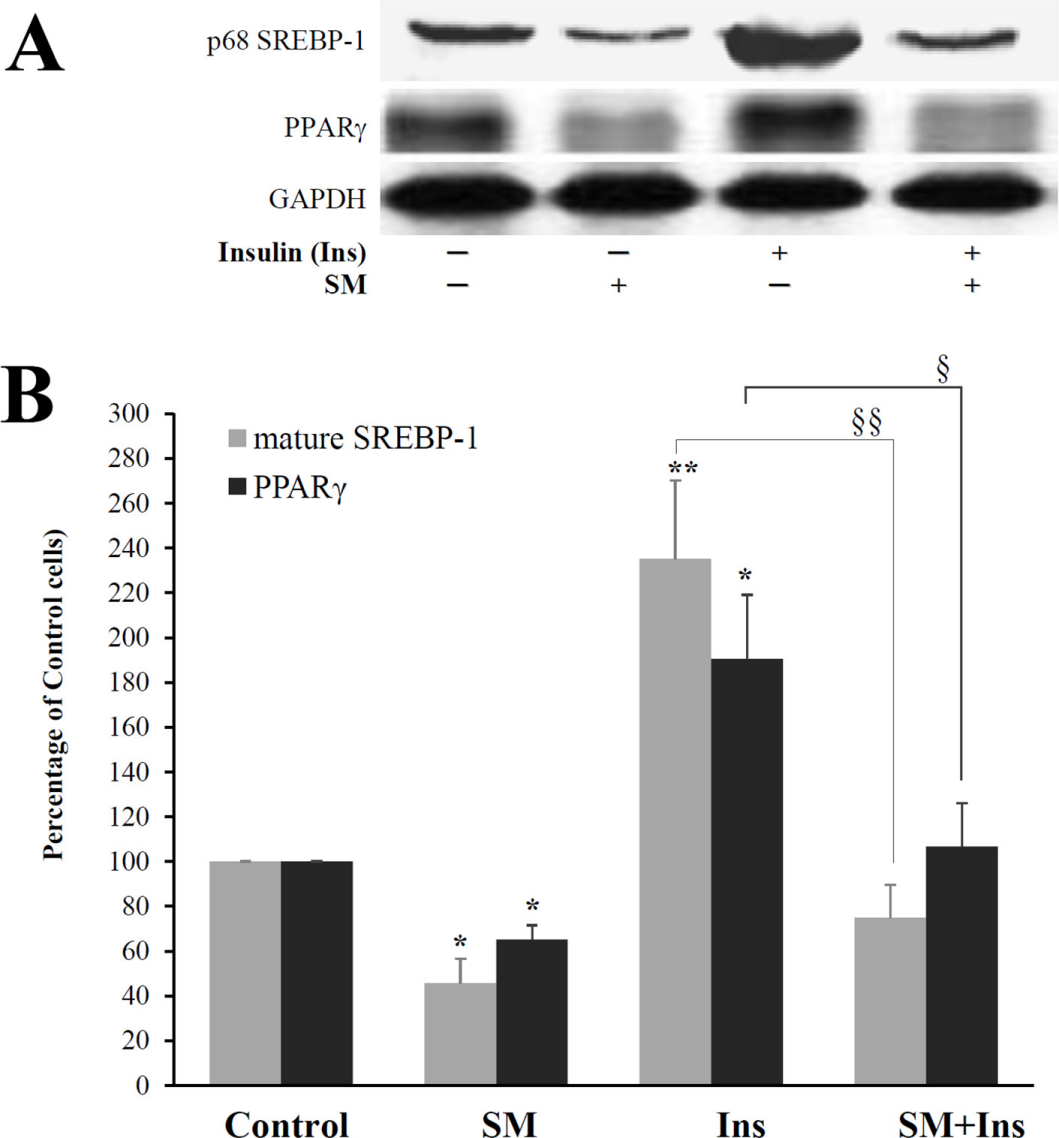
SM inhibits the insulin-induced expression of SREBP-1. Differentiated adipocytes were treated with SM-LA (30 μM, 24 h) in the presence or absence of insulin (100 nM, 24 h). Immunoblot analysis of mature SREBP-1 proteins; Nuclear extracts were separated by SDS-PAGE (30 μg of protein) and immunoblotted with the indicated antibodies. Representative blots (A) and quantitative variations (B) are shown. The results are expressed as percentages of control cells and are the mean±SEM of five independent experiments, performed in duplicate. ^*^ *P*<0.05; SM compared to control. ^**^ *P*<0.01; Ins compared to control. ^§^ *P*<0.01 and ^§§^ *P*<0.005; SM+Ins compared to Ins.

Finally, the insulin sensitivity of the cells was examined whether it is affected by SM accumulation. The expression of nuclear SREBP-1 protein as well as PPARγ in response to insulin was measured in SM-enriched adipocytes (SM-LA 24 h, 30 μM) and control (cells treated with vehicle). As expected, insulin induced the expression of mature SREBP-1 (235%, *P*<0.01) and PPARγ (191%, *P*<0.05) in control SM-untreated cells. However, there was no stimulation in SM-treated cells (Fig 13).

## Discussion

We previously reported that excess sphingomyelin in the membranes of 3T3-F442A adipocytes decreased PPARγ expression [14] and that the SM/CHOL ratio represented an important determinant of glucose transport [32] in preadipocytes. These findings led us to further study the effect of SM overabundance on the expression of the transcription factor SREBP because SREBP-1 is a key regulator of glucose metabolism. SM accumulates in the plasma membrane within 24 h involving caveolae, followed by its subcellular distribution into intracellular compartments. SM affects SREBP expression via a MAP kinase-dependent mechanism. The correlations found in human samples support the findings.

The state of decreased membrane fluidity reflects the accumulation of SM in membranes. SM up-regulates SREBP-2 and caveolins affecting the subcellular distribution of CHOL. The data support the hypothesis that SM enrichment affects the CHOL compartmentalization, preferentially intracellular without its parallel accumulation in the plasma membranes (Fig 9); the rise in intracellular CHOL content of adipocytes (microscopic observation by filipin staining of the cells or quantitative evaluation in triglyceride droplets (~2 fold)) might most likely be attributable to an increase in intracellular triglycerides droplets-associated CHOL [53]. A perturbation of SM content alters CHOL synthesis, transport and balance [54]. Furthermore, the current data demonstrate that SM-enriched adipocytes have elevated SREBP-2 mRNA and protein levels, which is consistent with the activation of CHOL biosynthetic genes [17]. In addition, the expressions of Cav-1 and Cav-2 were elevated in SM-enriched adipocytes. The up-regulation of caveolin, the CHOL-binding protein [55], corresponds to a homeostatic response to readjust the sphingomyelin to CHOL ratio in adipocyte membranes and keep the plasma membrane microdomain assemblies intact. Caveolin up-regulation has been reported in CHOL-depleted 3T3-L1 adipocytes treated with compactin, which has been previously demonstrated to induce a slight increase in sphingomyelin in human macrophages [56,57].

Our data also indicate that in SM-unmodulated adipocytes treated with PPMP, where the ceramide level increased, the expression levels of SREBPs, caveolins, ERK and membrane fluidity were modulated in an opposite direction relative to the SM-enriched cells. This reveals a distinct role of SM and ceramide. Despite the opposite responses, accumulations of ceramide for both SM- and PPMP-treated cells were observed. However, glucosylceramide levels increased only in the case of SM-treated cells, unlike the PPMP-treated cells, where glucosylceramide levels decreased, indicating that glucosylceramide could have a potential synergetic effect in SM-modulated cells (Fig 11). In human hepatocytes [58], TNF-α, SMase, and C_2_-ceramide treatments increased the levels of endogenous ceramide and SREBP-1; in the present study, SREBP-1 expression increased in adipocytes treated with PPMP. In cultured CHO cells [59], sphingomyelin depletion inhibited SREBP-2 maturation; the converse is most likely also true, i.e., sphingomyelin excess up-regulates SREBP-2 maturation. The differential regulation of SREBP-1 and SREBP-2 by SM is compatible with previous data suggesting that the increase in SREBP-2 expression occurs at the expense of SREBP-1 expression [60]. Here, PD98059, which inhibits ERK1/2 phosphorylation, down-regulated SREBP-1 expression but up-regulated SREBP-2 expression (Fig 6).

Our study supports the concept that SM controls SREBP-1 by regulating ERK through a MAPK pathway involving caveolin. It has been previously reported that SMase and ceramide activates MAPK and the inhibition of the N-SMase leads to the inhibition of ERK [61,62]. SM down-regulates Ras/Raf/MEK and KSR proteins, which are upstream mediators of ERK. The MAP kinase cascade can also be activated by certain heterotrimeric G proteins; most of these proteins require Ras [63]. Interestingly, the accumulation of SM in the caveolae fraction (~6.5-fold) reflects a dysregulation of the membrane and the MAPK pathway. Earlier observations by Galbiati *et al*. [20] revealed that caveolin functions as a negative regulator of the Ras-p42/44 MAPK cascade through a direct interaction with MEK/ERK. ERK, which localizes to caveolae, is initially inactive, and SM can prevent its activation by direct interaction and/or via an up-regulation of caveolin. Erk phosphorylation precedes changes in SREBP-1 [64]. SREBP-2 does not contain Erk phosphorylation sites [65].

The down-regulation of SREBP-1 by SMs could affect target genes, particularly caveolin and PPARγ. SREBP-1 has been previously identified as a transcriptional regulator of caveolin expression. The Cav-1 gene promoter contains two sterol regulatory (SRE)-like elements, and previous findings suggest that SREBP inhibits caveolin gene transcription [66]. Two SRE-like sequences were also identified in the Cav-2 gene promoter region. Therefore, the decrease in the mature form of SREBP-1 could explain the increased mRNA and protein levels of caveolins in SM-enriched adipocytes. Ectopic expression of ADD-1/SREBP-1 in 3T3-L1 and HepG2 cells induced endogenous PPARγ mRNA levels through direct binding to a PPARγ consensus E-box motif [48]; alternatively, SREBP-1 could activate PPARγ through the production/secretion of molecules that can act as ligands for PPARγ [67].

We observed that rosiglitazone promoted the maturation of SREBP-1, which is associated with the phosphorylation of CREB and the up-regulation of PPARγ expression in adipocytes, regardless of whether SM was present, indicating that rosiglitazone reversed the effects of SM on SREBP-1, PPARγ and CREB. The negative linear correlations found in human adipose tissues support the effect of SM on these targets. Our data show that co-treating the cells with rosiglitazone and SM did not inhibit the targets SREBP-1, PPARγ and CREB upon activation by thiazolidinedione. These results confirmed that SM altered this studied pathway at a step preceding PPARγ activation. PPARγ is considered a positively regulated SREBP-1 target gene [48], and CREB is an upstream co-regulator of SREBP-1 [49]. The activation of ERK is required for the regulation of CREB possibly by the recruitment of a coactivator, such as CBP [68]. Furthermore, CREB has been shown to promote insulin resistance in obesity [69] and to induce adipogenesis [70], whereas the expression of a dominant-negative form of CREB was shown to block adipogenesis in 3T3-L1 cells [70]. Our data indicates that insulin sensitivity was also affected by SM accumulation: SM inhibited insulin-stimulated expression of the nuclear SREBP-1 protein.

**Fig 14:**
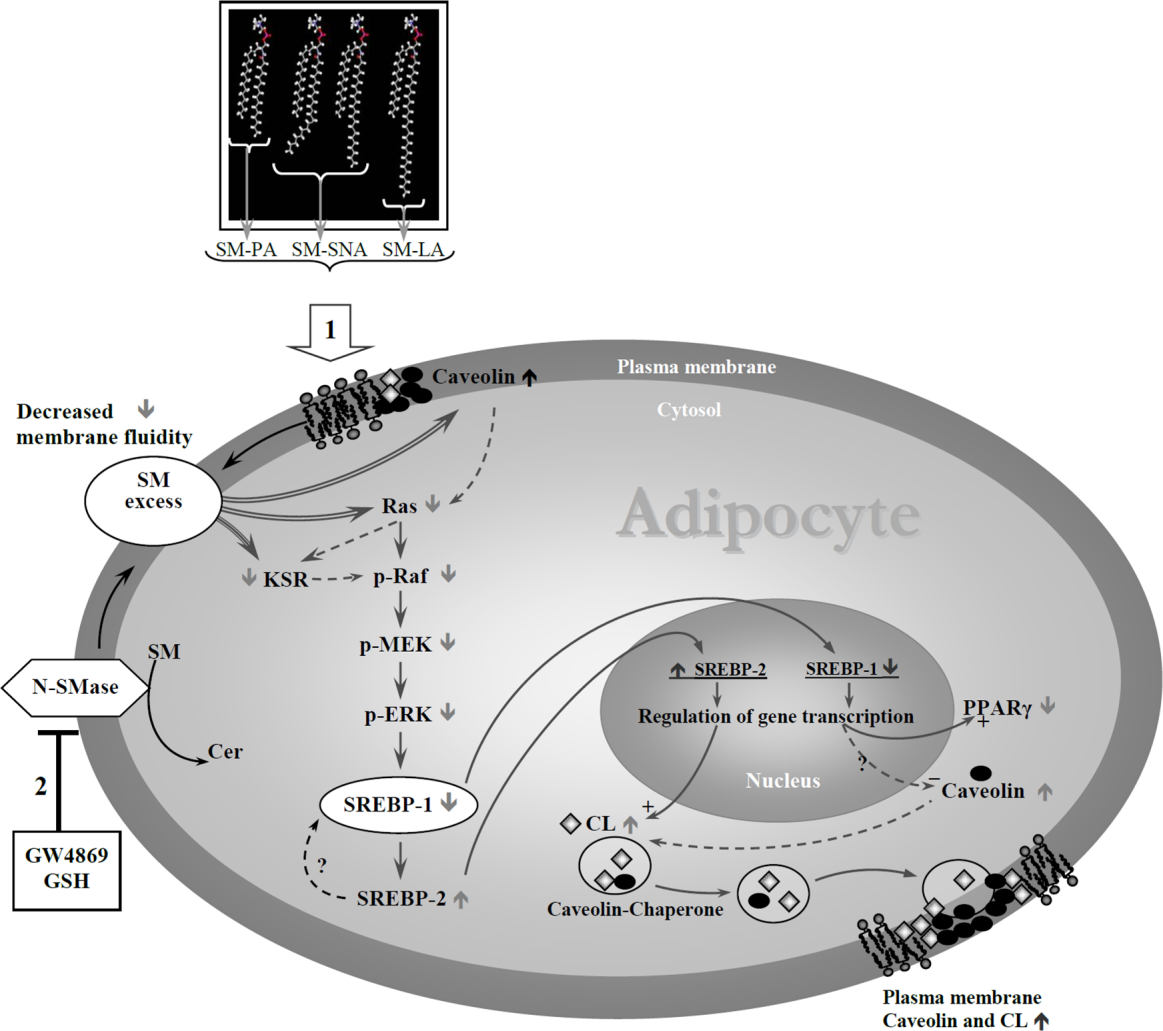
Hypothetical model depicting how sphingomyelin might regulate SREBPs in 3T3-F442A adipocytes. Illustration of how an SM-initiated signal transduction pathway leads to SREBP modulation. Two cell models of membrane SM enrichment were investigated in this report (1) by adding exogenous sphingomyelins in the culture medium of differentiated cells or (2) by inhibiting N-SMase with GW4869 or GSH. In turn, SM contents increase in membranes (caveolae). Membrane SM accumulation promotes a rigid state of the membrane and caveolin accumulation and regulates the Ras-Raf-ERK MAP kinase pathway, inhibiting SREBP-1. By inhibiting KSR, which is the putative target of ceramide, SM acts to amplify the signal provided to the Raf/ERK MAP kinase cascade. In turn, by affecting SREBP-1, SM induces SREBP-2. The increased levels of SREBP-2 are consistent with increased CHOL synthesis. In SM-enriched adipocytes, a higher amount of CHOL is required to maintain raft and caveolae assembly. Caveolin transports the neo-synthesized CHOL toward the cell surface, thus increasing the caveolin-2 levels in the plasma membrane. In SM-depleted cells, the opposite mechanism could be effective.

Finally, we propose a model (Fig 14) that incorporates these data and suggest a mechanism by which SM enrichment could initiate the regulation of SREBP-1 involving a MAPK pathway and underline the role of caveolin.

A body of evidence has suggested that sphingomyelin is an important lipid in pathophysiological processes [3,71–73]. In the present work and in our previous studies, we have demonstrated that excess membrane SM in adipocytes can down-regulate SREBP-1, PPARγ, CREB and Ras/Raf/MEK/ERK and up-regulate SREBP-2 and caveolins gene expression. These data suggest that SM could be a contributor to the dysregulation of genes in obesity. Currently, central obesity is thought to be the key factor that predisposes individuals to insulin resistance, metabolic syndrome X, and type 2 diabetes mellitus and is associated with a greater risk of cardiovascular diseases. The dysregulation of gene expression, namely, SREBPs and PPARγ, has been previously reported in these pathologies, particularly in syndrome X [74]. Caveolins have emerged as key players in shifting the focus of obesity and insulin resistance development to lipid dynamics and membrane microdomain disorders. Previous reports showed up-regulation of mRNA Cav-1 expression levels in visceral and subcutaneous adipose tissue of obese patients [75] and highlighted the role of caveolins in energy and metabolic homeostasis as well as in body weight and insulin resistance [76,77].
